# Adaptive mutations at lysine residues of PRRSV-2 nsp12 enable evasion of host proteasomal degradation to promote subgenomic RNA synthesis

**DOI:** 10.1128/jvi.00479-26

**Published:** 2026-06-10

**Authors:** Yongjie Chen, Zishen Chen, Ling Huang, Siyong Zeng, Baoying Huang, Chunhe Guo

**Affiliations:** 1 Guangdong Laboratory for Lingnan Modern Agriculture, State Key Laboratory of Animal Disease Control and Prevention, Key Laboratory of Zoonosis Prevention and Control of Guangdong Province, College of Veterinary Medicine,South China Agricultural University 12526, Guangzhou, Guangdong, People's Republic of China; University of Minnesota Twin Cities, Minneapolis, Minnesota, USA

**Keywords:** PRRSV-2, nsp12, proteasomal degradation, sgRNA synthesis, adaptive mutations

## Abstract

Ubiquitin signaling in viral infections can either enhance immune responses and degrade viral proteins or be exploited by viruses to target host antiviral factors. Porcine reproductive and respiratory syndrome virus (PRRSV) poses a threat to the swine industry, with its non-structural proteins (nsps) critical for virulence and replication. Here, we investigated the role of ubiquitination in regulating the stability of PRRSV-2 nsps and found that nsp12 was specifically targeted for degradation via the ubiquitin-proteasome system. Mechanistically, nsp12 underwent K48- and K63-linked polyubiquitination at lysine residues 89, 91, 127, and 130. RNF114 served as an E3 ubiquitin ligase for nsp12, and its enzymatic activity was essential for both nsp12 degradation and viral replication. Notably, evolutionary analyses revealed that PRRSV-2 nsp12 acquired lysine-to-arginine substitutions at positions 89, 127, and 130 during viral adaptation, which facilitated the synthesis of subgenomic RNA. Consistently, recombinant PRRSV-2 carrying the K91/127/130R mutations within nsp12 exhibited enhanced replication, while a revertant strain with the R89K mutation in nsp12 showed attenuated infectivity. Mass spectrometry analysis further identified significant enrichment of ubiquitination-related modifications among nsp12-interacting proteins. These findings provide valuable insights for anti-PRRSV-2 drug design and highlight the challenge posed by adaptive mutations in viral proteins to the swine industry.

**IMPORTANCE:**

Ubiquitin-mediated proteasomal degradation is a central antiviral mechanism; however, how porcine reproductive and respiratory syndrome virus (PRRSV) counteracts this host defense has remained unclear. Here, we demonstrate that PRRSV-2 has evolved a strategy to escape this restriction. We show that while the viral non-structural protein 12 (nsp12) is normally targeted for degradation by ubiquitination, adaptive mutations that eliminate key ubiquitination sites allow the virus to evade proteasomal clearance. This escape not only stabilizes nsp12 but also promotes the synthesis of viral subgenomic RNA, thereby enhancing viral replication. Our findings reveal a novel immune evasion mechanism in which a virus acquires specific mutations to subvert ubiquitin-dependent host defenses, highlighting a critical challenge for the development of effective vaccines and antivirals against PRRSV-2.

## INTRODUCTION

Livestock farming is a crucial pillar of the global food supply chain; however, bacterial and viral diseases pose challenges to its development (1). These diseases not only lead to animal mortality and reduced production performance, resulting in substantial economic losses, but also pose potential threats to food safety (2, 3). Since its first report in the United States in 1987, porcine reproductive and respiratory syndrome (PRRS) has spread worldwide, including in China, severely impeding the development of the pig farming industry and causing significant economic losses (4, 5). PRRS virus (PRRSV) is an enveloped, single-stranded positive-sense RNA virus belonging to the family *Arteriviridae* and the genus *Betaarterivirus*. It comprises two genetically distinct species: PRRSV-1 (*Betaarterivirus suid 1*) and PRRSV-2 (*Betaarterivirus suid 2*). Despite sharing only ~60% sequence identity, both elicit similar clinical syndromes in pigs (6, 7). The genome of PRRSV is approximately 15 kb in length and contains at least 11 open reading frames (ORFs), encoding at least 16 non-structural proteins (nsps) and 8 structural proteins (8, 9). Pigs of all ages are susceptible to PRRSV, with pregnant sows and newborn piglets being the most susceptible (10). PRRSV primarily infects porcine macrophages, particularly porcine alveolar macrophages (PAMs). After replicating within macrophages, the virus disseminates to lymphoid tissues and the lungs (11). It causes severe reproductive disorders in sows, respiratory diseases in piglets, and secondary infections (12). As an RNA virus, PRRSV evolves rapidly due to its high mutation and recombination rates, enabling it to generate new mutant strains quickly (13). Genomic variation allows PRRSV to easily evade host defense mechanisms and immune system recognition, posing significant challenges for epidemic prevention and control (14).

In recent years, with the growing threat posed by emerging pathogens to public health security and the lack of effective drugs and vaccines for certain traditional pathogens, finding new therapeutic targets has become a global research focus (15, 16). In this context, ubiquitination has gradually gained widespread attention. As a crucial posttranslational modification, ubiquitination plays vital roles in regulating protein activity, protein-protein interactions, and subcellular localization (17). It not only governs the function of individual proteins but also participates extensively in the regulation of nearly all cellular processes, including the cell cycle, proliferation, apoptosis, differentiation, signal transduction, tissue repair, inflammation, and immune responses (18–20). With the further advances in ubiquitination research, its roles in host immunity and viral infection have gradually garnered attention. Ubiquitination regulates both innate and adaptive immune responses by modulating the activation, proliferation, and differentiation of immune cells, as well as the production and secretion of immune mediators (20, 21). Viruses often hijack the host ubiquitination system to evade immune surveillance, tune the stability and activity of viral proteins, and facilitate viral assembly and release, thereby promoting viral replication and dissemination (22–24). Conversely, the host employs ubiquitination to modify viral proteins, targeting key viral proteins or host factors usurped by viruses for degradation, thereby restricting viral infection and replication (25, 26). Despite extensive progress in understanding ubiquitination in viral infections, whether and how PRRSV manipulates or is modulated by ubiquitination remains largely unclear.

In this study, we found that the nsp12 of PRRSV-2 was unstable in host cells; it underwent polyubiquitination and was subsequently degraded via the ubiquitin-proteasome pathway. To evade these host defense mechanisms, PRRSV-2 had acquired adaptive mutations at lysine residues within nsp12 during genetic evolution, thereby enhancing its resistance to host-mediated degradation and facilitating the synthesis of viral subgenomic RNA (sgRNA) and more efficient viral replication. Our research not only reveals the crucial role of the ubiquitin-proteasome system in host antiviral defense but also elucidates how viruses escape these defenses through adaptive evolution.

## RESULTS

### PRRSV-2 nsp12 undergoes degradation via the ubiquitin-proteasome pathway

**Fig 1:**
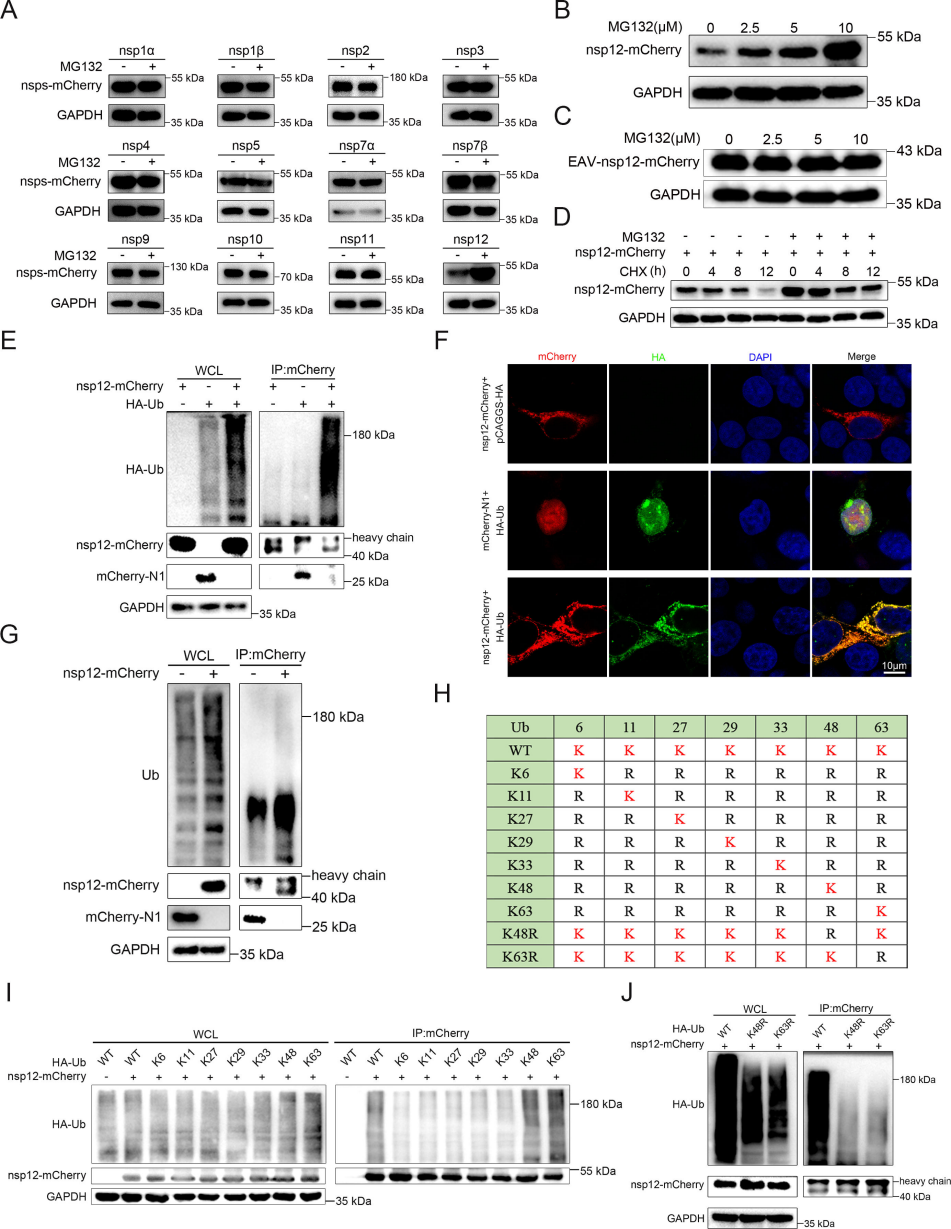
PRRSV-2 nsp12 undergoes degradation via the ubiquitin-proteasome pathway. (**A**) HEK293T cells were transfected with PRRSV-2 nsps-mCherry plasmids and treated with MG132 (10 μM) or DMSO for 12 h prior to harvest. Protein levels of nsps were detected by western blotting. (**B**) HEK293T cells were transfected with nsp12-mCherry and treated with different concentrations of MG132 for 12 h before harvesting. Protein levels of nsp12 were analyzed by western blotting. (**C**) HEK293T cells were transfected with EAV-nsp12-mCherry and treated with different concentrations of MG132 for 12 h before harvesting. Protein levels of EAV-nsp12-mCherry were analyzed by western blotting. (**D**) HEK293T cells were transfected with the nsp12-mCherry for 12 h, then treated with or without MG132 (10 μM) for 12 h, followed by the addition of CHX (50 μg/mL). Cells were harvested at different time points, and the cell lysates were analyzed. (**E and F**) HEK293T cells were transfected with nsp12-mCherry or an empty vector, along with HA-Ub or an empty vector. Co-IP was performed using anti-mCherry beads, followed by western blotting (**E**). Confocal assays were used to assess the colocalization of nsp12 and Ub (**F**). (**G**) HEK293T cells were transfected with nsp12-mCherry alone. Whole cell lysates (WCL) were incubated with anti-mCherry beads and subjected to western blotting. (**H**) Schematic diagram of Ub mutants (K6, K11, K27, K29, K33, K48, K63, K48R, and K63R). (**I and J**) HEK293T cells were transfected with nsp12 and Ub-WT or Ub mutants. Co-IP was performed using anti-mCherry beads, followed by western blotting. All experiments were repeated at least three times with independent biological replicates.

To investigate whether the stability of PRRSV-2 nsps is regulated by the ubiquitin-proteasome pathway, we examined the effect of the proteasome inhibitor MG132 on the viral nsps abundance. MG132 treatment specifically increased nsp12 protein levels in a concentration-dependent manner in both HEK293T and 3D4/21 cells but had no such effect on other nsps (Fig. 1A and B; Fig. S1A). This effect appeared specific to PRRSV-2, as the degradation of nsp12 from the related arterivirus equine arteritis virus (EAV) was not rescued by MG132 (Fig. 1C). Furthermore, cycloheximide (CHX) treatment resulted in the rapid degradation of nsp12, while MG132 treatment stabilized its protein levels (Fig. 1D; Fig. S1B). These results demonstrate that nsp12 is unstable and can be degraded via the proteasome pathway.

As ubiquitination is a prerequisite for proteasomal degradation, we next determined whether nsp12 undergoes ubiquitination. Co-immunoprecipitation (Co-IP) assays confirmed that nsp12 was modified with polyubiquitin chains (Fig. 1E; Fig. S1C). Ubiquitin also relocalized from the nucleus to the cytoplasm, where it colocalized with nsp12 (Fig. 1F). Additionally, endogenous ubiquitin was found to bind to nsp12 (Fig. 1G), further verifying nsp12 ubiquitination in cells. To identify the ubiquitin linkage types, we coexpressed nsp12 with wild-type ubiquitin (HA-Ub-WT) or lysine-specific mutants (K6, K11, K27, K29, K33, K48, K63, K48R, and K63R). Nsp12 was specifically modified by K48- and K63-linked polyubiquitination (Fig. 1H and I), a result confirmed in 3D4/21 cells (Fig. S1D). In contrast, cotransfection with the HA-Ub-K48R and HA-Ub-K63R plasmids reduced nsp12 ubiquitination (Fig. 1J). Together, these findings confirm that nsp12 is modified by K48- and K63-linked ubiquitin chains, which target it for proteasomal degradation.

### Ubiquitin-proteasome degradation of PRRSV-2 nsp12 via K89/K91/K127/K130 ubiquitination

**Fig 2:**
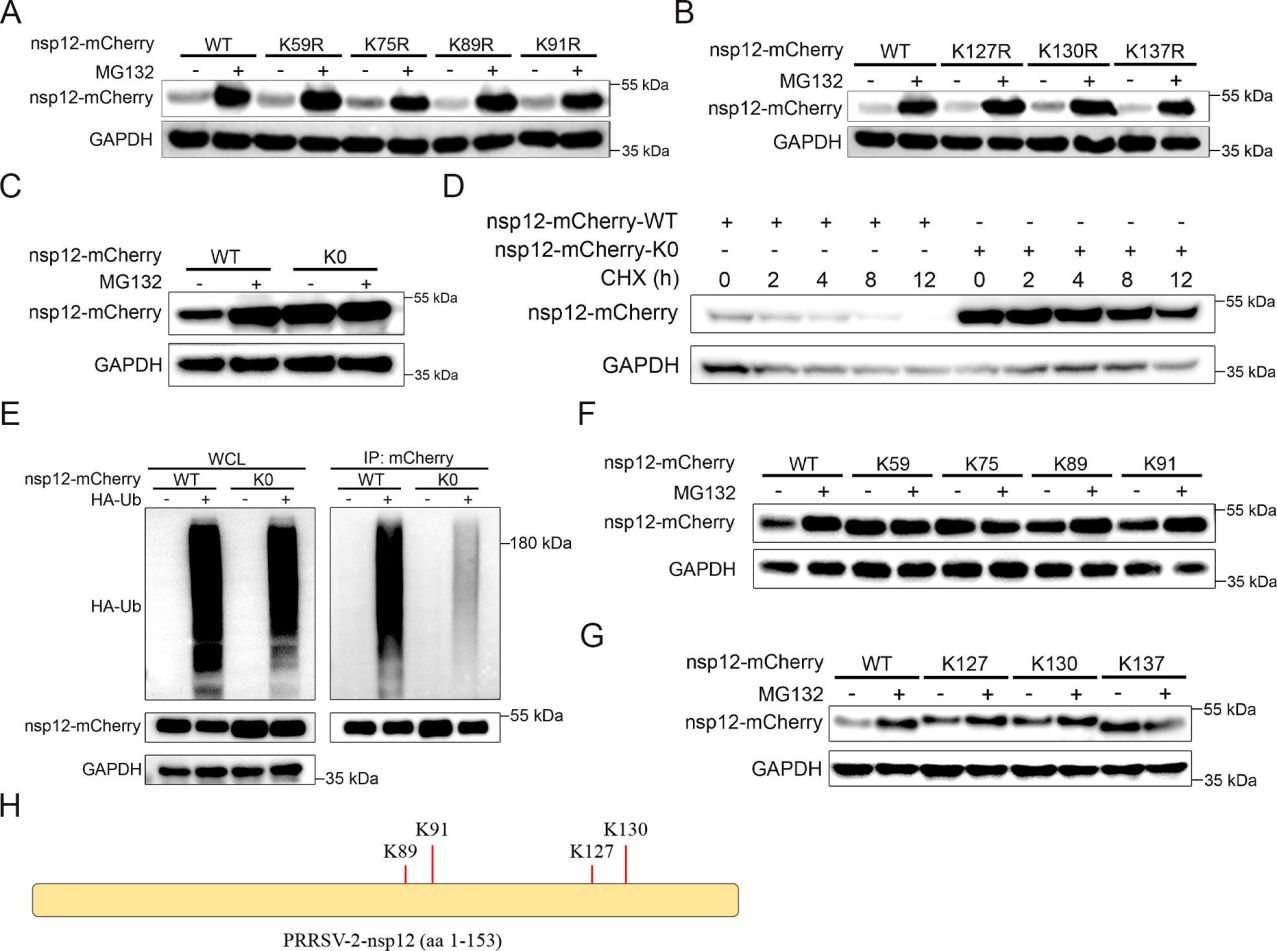
Ubiquitin-proteasome degradation of PRRSV-2 nsp12 via K89/K91/K127/K130 ubiquitination. (**A and B**) HEK293T cells were transfected with nsp12 or nsp12 lysine mutants and treated with or without MG132 (10 μM) for 12 h prior to harvest and analysis by western blotting. (**C**) HEK293T cells were transfected with nsp12-WT or nsp12-K0 and treated with or without MG132 (10 μM) for 12 h prior to harvest and analysis by western blotting. (**D**) HEK293T cells were transfected with nsp12-WT or nsp12-K0 for 24 h, followed by the addition of CHX (50 μg/mL). Cells were harvested at different time points, and the cell lysates were analyzed. (**E**) HEK293T cells were transfected with nsp12-WT or nsp12-K0, along with HA-Ub or an empty vector. Co-IP was performed using anti-mCherry beads, followed by western blotting. (**F and G**) HEK293T cells were transfected with nsp12-WT or nsp12 single mutants, where arginine was replaced by lysine based on the K0 mutant. Cells were treated with or without MG132 (10 μM) for 12 h prior to harvest and analysis by western blotting. It should be noted that the nsp12^K127^-mCherry and nsp12^K130^-mCherry mutants in panel G exhibit a slightly higher molecular weight. This is due to the plasmid construction strategy, which retained additional vector-derived amino acids, thus increasing their apparent size. (**H**) Schematic diagram of nsp12 ubiquitination sites. All experiments were repeated at least three times with independent biological replicates.

To further identify the ubiquitination sites on nsp12, we analyzed its full-length sequence and identified seven lysine residues. These residues were individually mutated to arginine to assess their role in nsp12 ubiquitination (Fig. S2A). Treatment with MG132 increased the expression levels of all nsp12 mutants, indicating that multiple lysine residues contribute to nsp12 ubiquitination and subsequent degradation (Fig. 2A and B). We next generated a K0 mutant by mutating all seven lysine residues to arginine. In contrast to nsp12-WT, MG132 treatment did not increase the protein levels of the nsp12-K0 mutant (Fig. 2C). When cells overexpressing nsp12-WT or nsp12-K0 were treated with CHX, nsp12-K0 mutants exhibited higher protein stability than nsp12-WT (Fig. 2D). Furthermore, the ubiquitination levels of nsp12-K0 were nearly undetectable compared to nsp12-WT (Fig. 2E). To identify specific ubiquitination sites, we individually restored each lysine residue in the nsp12-K0 mutant backbone (Fig. S2B). The results showed that the protein levels of the nsp12^K89^, nsp12^K91^, nsp12^K127^, and nsp12^K130^ mutants were reduced but stabilized upon MG132 treatment (Fig. 2F and G). Notably, MG132 still increased the protein levels of the nsp12^K127/130R^. However, MG132 failed to increase the protein levels of the nsp12^K89/91/127/130R^ (Fig. S2C). CHX chase assays further confirmed that nsp12^K89/91/127/130R^ had higher protein stability than nsp12-WT (Fig. S2D). Additionally, nsp12^K89/91/127/130R^ exhibited fewer polyubiquitin chains compared to nsp12-WT (Fig. S2E). Collectively, these results demonstrate that nsp12 undergoes ubiquitin-proteasomal degradation mediated by ubiquitination at lysine residues 89, 91, 127, and 130 (Fig. 2H).

### RNF114 specifically ubiquitinates nsp12 at K127 and K130 in a manner dependent on its E3 ubiquitin ligase activity

**Fig 3:**
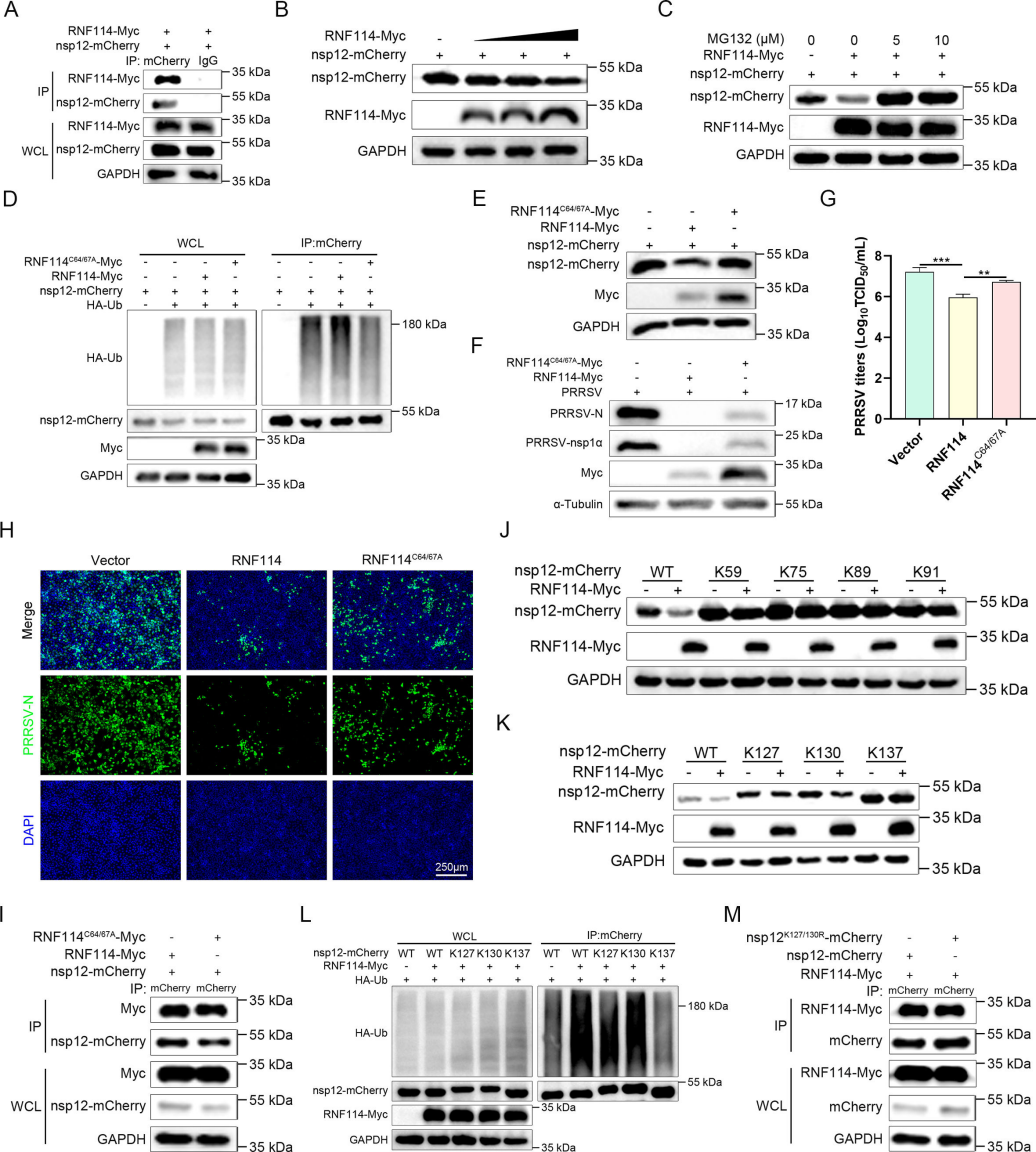
RNF114 specifically ubiquitinates nsp12 at K127 and K130 in a manner dependent on its E3 ubiquitin ligase activity. (**A**) HEK293T cells were cotransfected with nsp12 and RNF114. Co-IP was performed using anti-mCherry or anti-IgG beads, followed by western blotting. (**B**) HEK293T cells were cotransfected with nsp12 and increasing amounts of RNF114, followed by western blotting. (**C**) HEK293T cells expressing nsp12 with or without RNF114 were treated with the indicated concentrations of MG132 for 12 h before harvesting for western blotting. (**D**) HEK293T cells were cotransfected with nsp12 and either RNF114 or RNF114^C64/67A^, with or without HA-Ub. Co-IP was performed using anti-mCherry beads. (**E**) HEK293T cells were cotransfected with nsp12 and either RNF114 or RNF114^C64/67A^, followed by western blotting. (**F–H**) Marc-145 cells were transfected with RNF114 or RNF114^C64/67A^ for 24 h and then infected with PRRSV-2 strain CH-1a at a multiplicity of infection (MOI) of 0.5 for 24 h. The viral N and nsp1α protein levels were measured by western blotting (**F**). Viral titers in supernatants were quantified by TCID_50_ (**G**). N protein expression was assessed by IFA (**H**). (**I**) HEK293T cells were cotransfected with nsp12 and either RNF114 or RNF114^C64/67A^. Co-IP was performed using anti-mCherry beads. (**J and K**) HEK293T cells were transfected with nsp12-WT or single-lysine nsp12 mutants, with or without RNF114, followed by western blotting. (**L**) HEK293T cells were cotransfected with nsp12-WT or nsp12 mutants (nsp12^K127^, nsp12^K130^, or nsp12^K137^) along with HA-Ub and either RNF114 or an empty vector. Co-IP was performed using anti-mCherry beads. (**M**) HEK293T cells were cotransfected with RNF114 and either nsp12-WT or nsp12-K127/130R. Co-IP was performed using anti-mCherry beads. All experiments were repeated at least three times with independent biological replicates. Data are shown as mean ± SD (*n* = 3). *: *P* < 0.05; **: *P* < 0.01; ***: *P* < 0.001; ns: not significant (Student’s *t*-test).

Previous studies have shown that RNF114, an E3 ubiquitin ligase, interacts with nsp12 and promotes its proteasomal degradation (27), but the precise underlying mechanism remains unclear. In this study, we confirmed that nsp12 interacted with RNF114 and underwent RNF114-mediated degradation through the ubiquitin-proteasome pathway in HEK293T cells (Fig. 3A through C); this finding was recapitulated in 3D4/21 cells (Fig. S3A and B). Given the genetic diversity of PRRSV, we further assessed whether RNF114-mediated nsp12 degradation inhibits replication across different PRRSV-2 strains. Overexpression of RNF114 in cells subsequently infected with various PRRSV-2 strains (CH-1a, TA-12, JXA1, SD16, and WUH3) resulted in a dose-dependent reduction in viral N and nsp1α protein levels (Fig. S3C through G). Conversely, RNF114 knockdown in PAMs enhanced PRRSV-2 infection, as evidenced by increased viral mRNA expression and viral titers (Fig. S3H through K). These results indicate that RNF114 inhibits the replication of multiple PRRSV-2 strains.

We next investigated whether RNF114’s regulation of nsp12 depends on its E3 ligase activity. Mutation of the cysteine residues C64 and C67, which are essential for RNF114 ligase activity (28), abolished its ability to ubiquitinate nsp12 (Fig. 3D; Fig. S3L) and impaired nsp12 degradation in both HEK293T and 3D4/21 cells (Fig. 3E; Fig. S3M). Consequently, the catalytically inactive RNF114 mutant exhibited a reduced capacity to antagonize PRRSV-2 replication (Fig. 3F through H). Importantly, this mutant retained binding affinity for nsp12 comparable to wild-type RNF114 (Fig. 3I), confirming that the loss of antiviral function stems from impaired catalytic activity rather than disrupted nsp12 binding.

To map the ubiquitination sites on nsp12 mediated by RNF114, we mutagenized seven lysine residues. Only the nsp12^K127^ or nsp12^K130^ mutants remained susceptible to RNF114-induced degradation, whereas all other mutants were resistant (Fig. 3J and K). Consistently, RNF114 specifically enhanced nsp12 ubiquitination at K127 and K130, but not at K137 (Fig. 3L). Notably, the nsp12^K127/130R^ mutant still interacted with RNF114 (Fig. 3M), confirming that K127 and K130 are not required for the RNF114-nsp12 interaction but serve as the specific ubiquitination sites targeted by RNF114. Collectively, our study demonstrates that RNF114, via its E3 ubiquitin ligase activity, specifically ubiquitinates PRRSV-2 nsp12 at K127 and K130, thereby promoting nsp12 proteasomal degradation.

### Multiple lysine residues within nsp12 show a tendency to mutate to arginine during the genetic evolution of PRRSV-2

**Fig 4:**
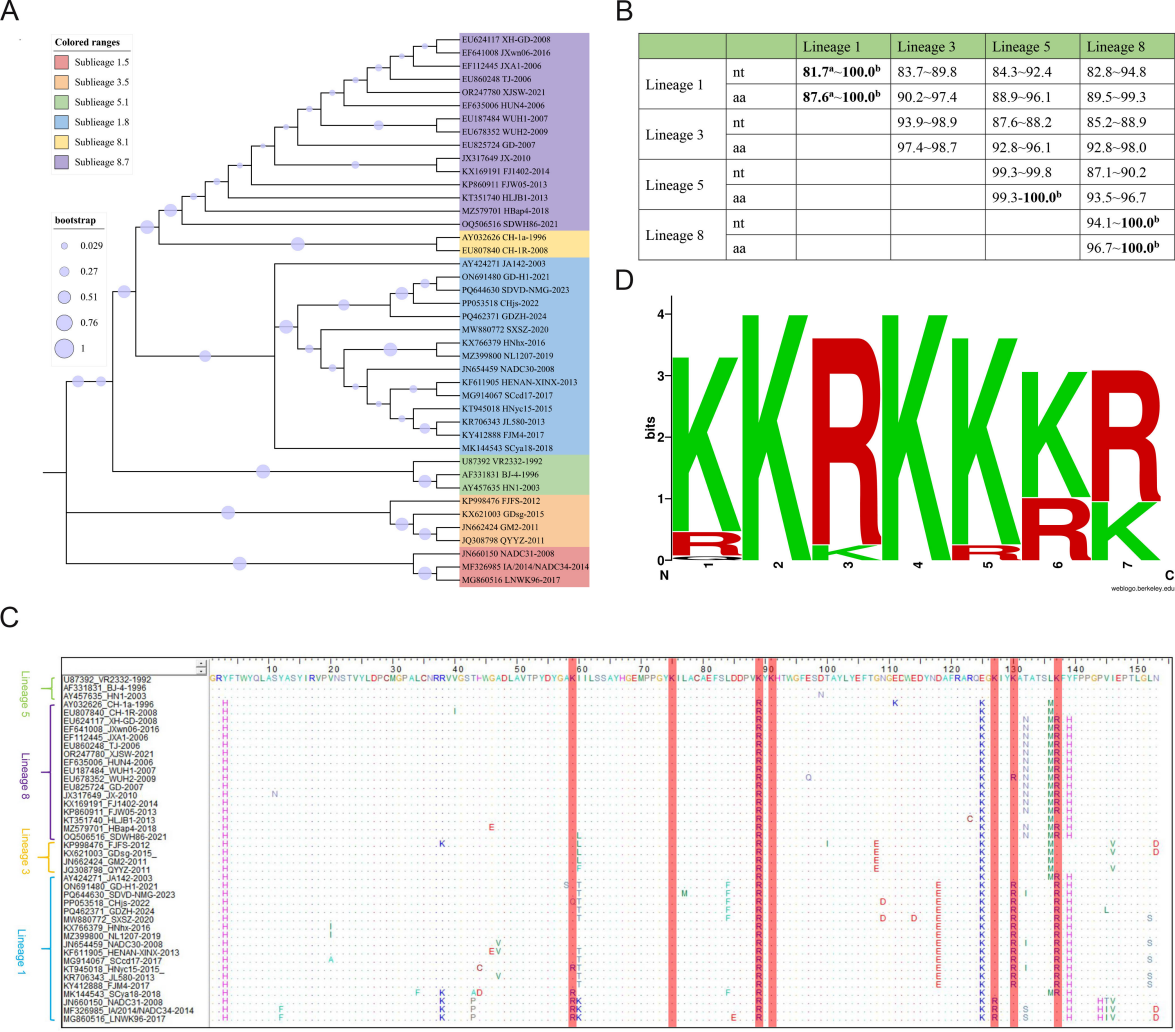
Multiple lysine residues within nsp12 show a tendency to mutate to arginine during the genetic evolution of PRRSV-2. (**A**) Phylogenetic analysis of nsp12. The phylogenetic tree was constructed using the NJ method in MEGA software with 1,000 bootstrap replicates. (**B**) Nucleotide and amino acid homology analysis based on the nsp12 (%). ^a^Indicates the lowest homology; ^b^Indicates the highest homology. (**C**) Alignment of nsp12 amino acid sequences from different PRRSV-2 strains. (**D**) The frequency of lysine mutations at positions 59, 75, 89, 91, 127, 130, and 137 in PRRSV-2 nsp12 was analyzed using the website (http://weblogo.berkeley.edu/).

To adapt to environmental pressures and evade host defenses, viral proteins often evolve adaptive mutations at key amino acid residues to promote viral survival and replication. To investigate whether PRRSV-2 nsp12 has evolved non-lysine substitutions to escape host ubiquitin-proteasome degradation, we performed phylogenetic analysis using nsp12 sequences retrieved from the GenBank database (Table S1). The results revealed that lineages 1 and 8 were the dominant currently circulating PRRSV-2 strains (Fig. 4A). Most strains deposited from 2020 to 2024 clustered within these two lineages, further supporting their epidemiological dominance (Fig. 4A). Comparative analyses of nsp12 nucleotide and amino acid homology across four major lineages revealed substantial genetic diversity. Lineage 1 displayed the highest level of diversity (nucleotide homology: 81.7%~100.0%; amino acid homology: 87.6%~100.0%), whereas lineage 5 was the most conservative (nucleotide homology: 99.3%~99.8%; amino acid homology: 99.3%~100.0%) (Fig. 4B). Importantly, relative to the ancestral strain VR-2332, we identified recurrent lysine-to-arginine substitutions at positions 59, 89, 127, 130, and 137 in contemporary field strains (Fig. 4C and D; Table S2). Notably, three of these lysine residues (K89, K127, and K130) corresponded to ubiquitination sites functionally validated in this study. These findings suggested that PRRSV-2 has evolved adaptive lysine-to-arginine substitutions at critical ubiquitination sites to escape host-mediated proteasomal degradation, thereby enhancing viral fitness.

To determine whether a similar evolutionary strategy exists in PRRSV-1, we compared nsp12 amino acid sequences between PRRSV-1 (Lelystad) and PRRSV-2 (VR-2332). The key lysine residues identified in PRRSV-2 (K89, K91, K127, and K130) were not conserved in PRRSV-1; instead, these positions were predominantly occupied by other amino acids (Fig. S4A). Moreover, no comparable adaptive lysine-to-arginine substitutions were observed within nsp12 of PRRSV-1 strains (Fig. S4B). Collectively, these results demonstrate that the evolution of site-specific lysine-to-arginine substitutions to evade proteasomal degradation is a unique adaptive feature of PRRSV-2, rather than a conserved mechanism shared by PRRSV-1 and PRRSV-2.

### The rPRRSV-nsp12^K91/127/130R^ exhibits greater replication capacity compared to rPRRSV

**Fig 5:**
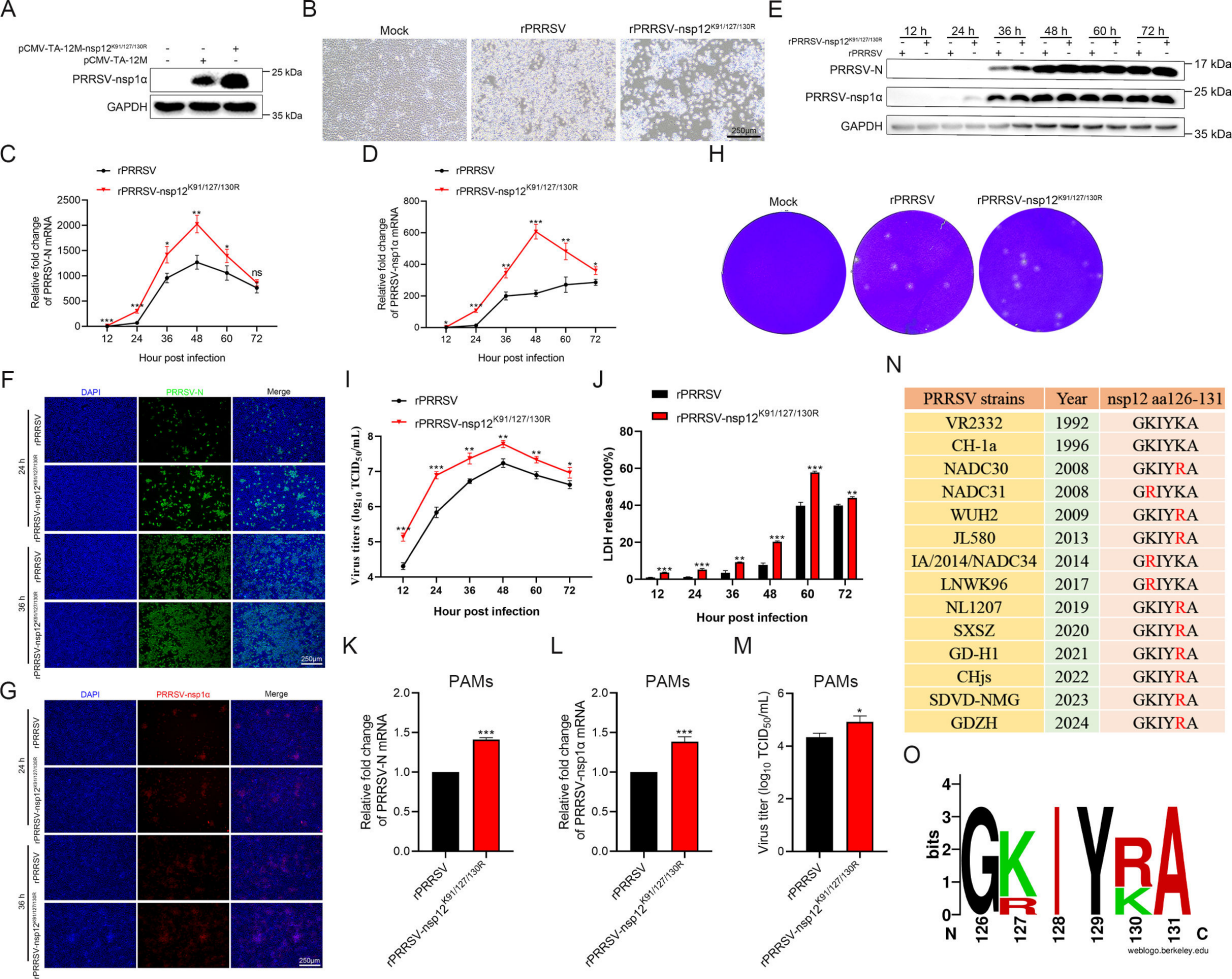
The rPRRSV-nsp12^K91/127/130R^ exhibits greater replication capacity compared to rPRRSV. (**A**) HEK293T cells in a six-well plate were transfected with 2.5 μg cDNA clone plasmid using Lipofectamine 3000. At 48 h, cells were harvested for nsp1α protein detection by western blotting. (**B**) Marc-145 cells in 12-well plates were infected with recombinant viruses at an MOI of 0.05 for 84 h. CPE was observed under a light microscope. (**C–E**) Marc-145 cells in 12-well plates were infected with recombinant viruses at an MOI of 0.05. The mRNA and protein levels of N and nsp1α were detected by RT-qPCR and western blotting. (**F and G**) Marc-145 cells in 12-well plates were infected with recombinant viruses at an MOI of 0.05 for 24 and 36 h. The expression of N and nsp1α proteins was assessed by IFA. (**H**) Marc-145 cells in 12-well plates were infected with recombinant viruses at an MOI of 0.05 for 24 h, followed by a plaque assay. (**I and J**) Marc-145 cells in 12-well plates were infected with recombinant viruses at an MOI of 0.05. Culture supernatants were collected at 12, 24, 36, 48, 60, and 72 h, and viral titers (**I**) as well as LDH release (**J**) were measured. (**K–M**) PAMs in 12-well plates were infected with recombinant viruses at an MOI of 1. Viral N and nsp1α mRNA levels were measured by RT-qPCR (**K and L**), and viral titers in supernatants were quantified by TCID_50_ assay in Marc-145 cells (**M**). (**N**) Alignment of the amino acid sequences of positions 126–131 within nsp12 from different PRRSV-2 strains. (**O**) The frequency of amino acid changes at positions 126–131 in PRRSV-2 nsp12 (panel N) was analyzed using the WebLogo3 online tool. All experiments were repeated at least three times with independent biological replicates. Data are shown as mean ± SD (*n* = 3). *: *P* < 0.05; **: *P* < 0.01; ***: *P* < 0.001; ns: not significant (Student’s *t*-test).

To investigate whether PRRSV-2 nsp12 evades host-mediated degradation through adaptive mutations, we compared the lysine residues of the ancestral VR2332 strain with the contemporary PRRSV-2 TA-12 strain. Notably, lysine residues at positions 91, 127, and 130 of nsp12 were highly conserved between the two PRRSV-2 strains (Fig. S5A). Since single mutations at these sites were previously shown to be insufficient for complete immune evasion, we generated a recombinant PRRSV-2 strain (rPRRSV-nsp12^K91/127/130R^) using the TA-12 strain as the backbone (Fig. S5B). Following viral rescue in HEK293T cells, western blot analysis at 48 h post-transfection revealed higher PRRSV-2 nsp1α expression in cells transfected with the triple mutant compared to wild-type controls (Fig. 5A), suggesting that these lysine-to-arginine mutations may alter viral gene expression. The successful rescue of rPRRSV-nsp12^K91/127/130R^ was confirmed by multiple approaches: cytopathic effect (CPE) observation (Fig. S5C), indirect immunofluorescence assay (IFA) (Fig S5D and E), western blotting (Fig. S5F), reverse transcription (RT)-PCR (Fig. S5G), and Sanger sequencing (Fig. S5H). Subsequently, we evaluated the impact of these lysine-to-arginine mutations on PRRSV-2 replication in Marc-145 cells. The recombinant rPRRSV-nsp12^K91/127/130R^ induced more pronounced CPE than rPRRSV (Fig. 5B). Additionally, compared to rPRRSV, rPRRSV-nsp12^K91/127/130R^ infection resulted in higher mRNA and protein levels of the N and nsp1α (Fig. 5C through E), as well as increased fluorescence intensity (Fig. 5F and G). Although both strains formed similar-sized plaques, rPRRSV-nsp12^K91/127/130R^ exhibited a higher viral titer than rPRRSV (Fig. 5H and I). Moreover, rPRRSV-nsp12^K91/127/130R^ infection led to greater lactate dehydrogenase (LDH) release compared to rPRRSV (Fig. 5J). A similar phenomenon was observed in PAMs, the target cells of PRRSV, as demonstrated by the increased mRNA levels of PRRSV-2 N and nsp1α, as well as higher viral titers, following infection with rPRRSV-nsp12^K91/127/130R^ (Fig. 5K through M). Importantly, we observed an increase in the proportion of strains with lysine-to-arginine mutations at positions 127 and 130 of nsp12 over time (Fig. 5N and O), suggesting that these adaptive mutations may contribute to the epidemic dominance of PRRSV-2 strains by enhancing nsp12 stability.

### The rPRRSV-nsp12^R89K^ strain exhibits lower replication capacity compared to rPRRSV

**Fig 6:**
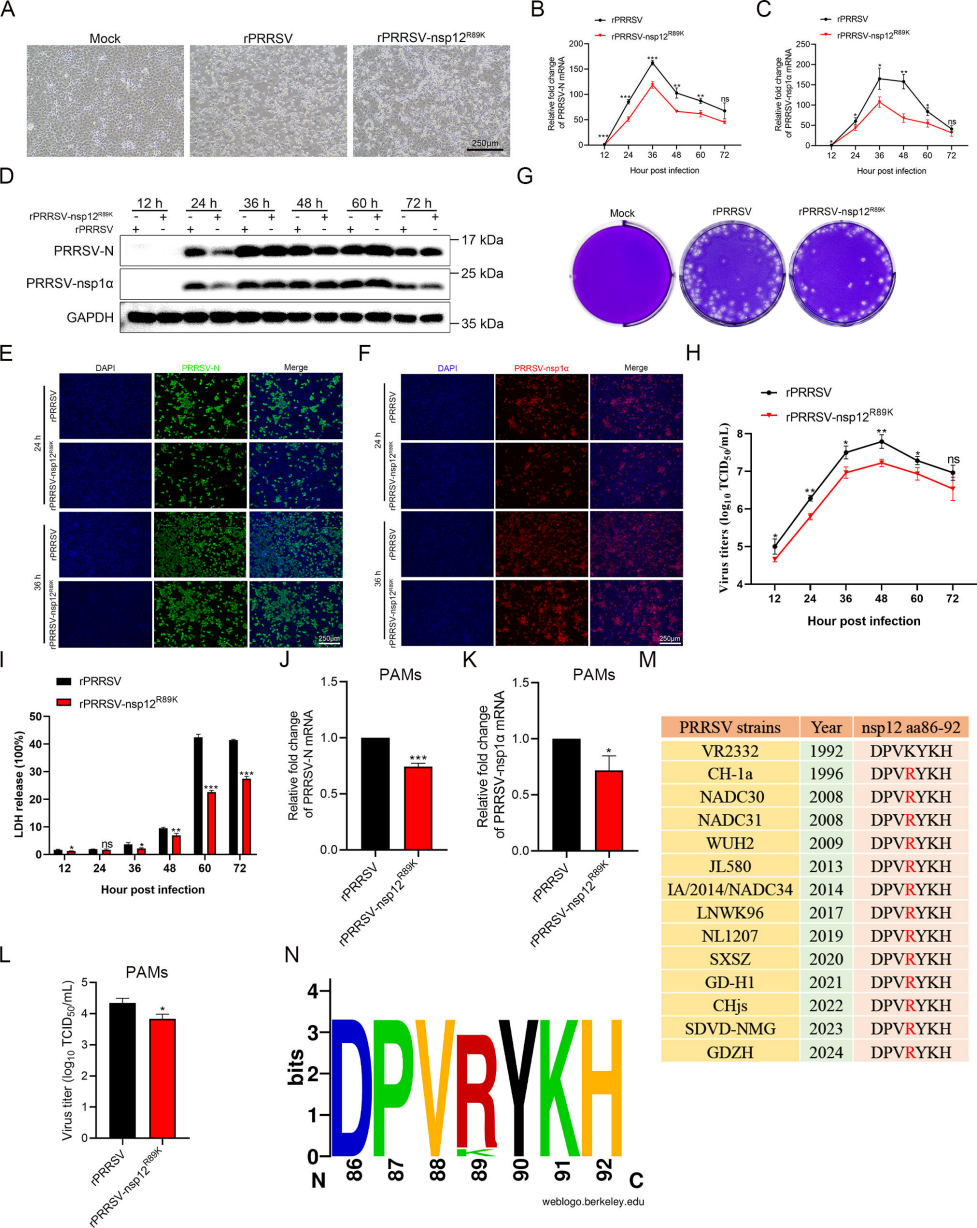
The rPRRSV-nsp12^R89K^ strain exhibits lower replication capacity compared to rPRRSV. (**A**) Marc-145 cells in 12-well plates were infected with recombinant viruses at an MOI of 0.1 for 78 h. CPE was observed under a light microscope. (**B–D**) Marc-145 cells in 12-well plates were infected with recombinant viruses at an MOI of 0.1. The mRNA and protein levels of N and nsp1α were detected by RT-qPCR and western blotting. (**E and F**) Marc-145 cells in 12-well plates were infected with recombinant viruses at an MOI of 0.1 for 24 and 36 h. The expression of N and nsp1α proteins was assessed by IFA. (**G**) Marc-145 cells in 12-well plates were infected with recombinant viruses at an MOI of 0.1 for 24 h, followed by a plaque assay. (**H and I**) Marc-145 cells in 12-well plates were infected with recombinant viruses at an MOI of 0.1. Culture supernatants were collected at 12, 24, 36, 48, 60, and 72 h, and viral titers (**H**) as well as LDH release (**I**) were measured. (**J–L**) PAMs in 12-well plates were infected with recombinant viruses at an MOI of 1. Viral N and nsp1α mRNA levels were measured by RT-qPCR (**J and K**), and viral titers in supernatants were quantified by TCID_50_ assay in Marc-145 cells (**L**). (**M**) Alignment of the amino acid sequences of positions 86–92 within nsp12 from different PRRSV-2 strains. (**N**) The frequency of amino acid changes at positions 86–92 in PRRSV-2 nsp12 (panel M) was analyzed using the Weblogo3 online tool. All experiments were repeated at least three times with independent biological replicates. Data are shown as mean ± SD (*n* = 3). *: *P* < 0.05; **: *P* < 0.01; ***: *P* < 0.001; ns: not significant (Student’s *t*-test).

Notably, sequence alignment revealed that the TA-12 strain harbored a K89R substitution in nsp12 compared to the prototype VR2332 strain (Fig. S5A). Given our previous demonstration that lysine at position 89 of PRRSV-2 nsp12 is a critical ubiquitination site, we hypothesized that reversion of this mutation (R89K) would restore ubiquitination-dependent degradation and attenuate viral replication. To test this hypothesis, we generated a recombinant virus (rPRRSV-nsp12^R89K^) based on the TA-12 strain backbone (Fig. S6A). The successful rescue of rPRRSV-nsp12^R89K^ was confirmed by CPE (Figure S6B), western blotting (Fig. S6C), IFA (Fig. S6D and E), RT-PCR (Fig. S6F), and Sanger sequencing (Fig. S6G). Further studies revealed that rPRRSV-nsp12^R89K^ induced weaker CPE compared to rPRRSV (Fig. 6A). Correspondingly, the mRNA (Fig. 6B and C) and protein (Fig. 6D) levels, as well as the fluorescence intensity (Fig. 6E and F) of N and nsp1α in rPRRSV-nsp12^R89K^, were lower than those in rPRRSV. Although both strains formed similar-sized plaques (Fig. 6G), the viral titer of rPRRSV-nsp12^R89K^ was lower than that of rPRRSV (Fig. 6H). Moreover, infection with rPRRSV-nsp12^R89K^ resulted in reduced LDH release compared to rPRRSV (Fig. 6I). Correspondingly, rPRRSV-nsp12^R89K^ exhibited lower levels of PRRSV N and nsp1α mRNA and reduced viral titers in PAMs compared to the wild-type virus (Fig. 6J through L). Strikingly, longitudinal sequence analysis of PRRSV-2 strains indicated that lysine at position 89 of nsp12 was replaced by arginine over time (Fig. 6M and N). These findings collectively demonstrate that the K89R substitution in PRRSV-2 nsp12 represents an evolutionary adaptation that enhances viral fitness by evading host ubiquitin-mediated defense mechanisms.

### Lysine-to-arginine adaptive mutations within nsp12 promote sgRNA synthesis

**Fig 7:**
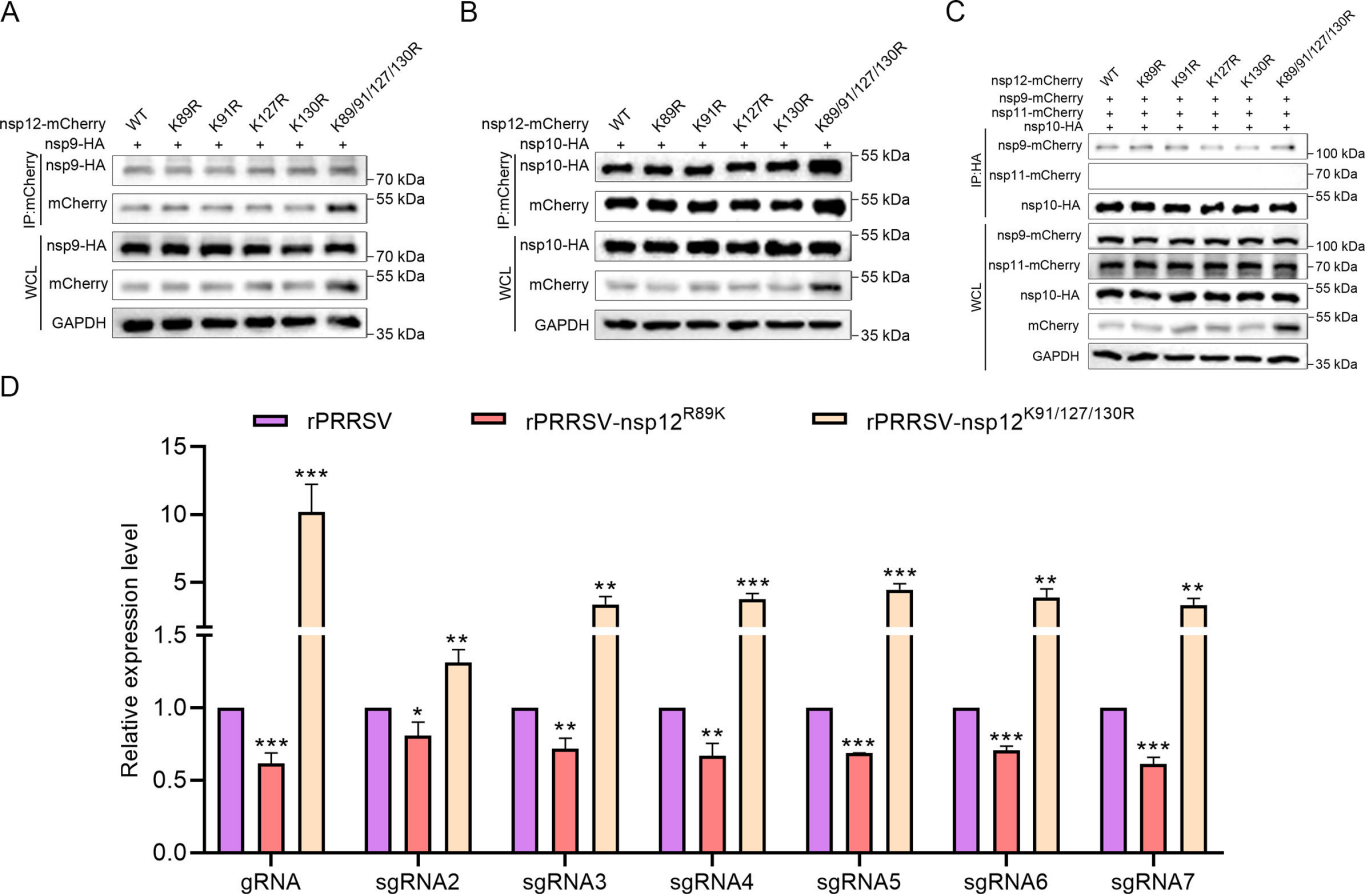
Lysine-to-arginine adaptive mutations within nsp12 promote sgRNA synthesis. (**A**) HEK293T cells were cotransfected with nsp12 (or its mutants) and nsp9. Cell lysates were then immunoprecipitated with anti-mCherry beads. (**B**) HEK293T cells were cotransfected with nsp12 (or its mutants) and nsp10. Cell lysates were then immunoprecipitated with anti-mCherry beads. (**C**) HEK293T cells were cotransfected with nsp12 (or its mutants) along with nsp9, nsp10, and nsp11. Cell lysates were then immunoprecipitated with anti-HA beads. (**D**) HEK293T cells in six-well plates were transfected with 2.5 μg cDNA clone plasmid using Lipofectamine 3000. At 48 h post-transfection, cells were harvested for RT-qPCR analysis. All experiments were repeated at least three times with independent biological replicates. Data are shown as mean ± SD (*n* = 3). *: *P* < 0.05; **: *P* < 0.01; ***: *P* < 0.001; ns: not significant (Student’s *t*-test).

Given the importance of lysine mutations in nsp12 for PRRSV-2 replication, we investigated whether these adaptive mutations affect the biological functions of nsp12. We first examined their impact on the interaction of nsp12 with nsp9 or nsp10. The results showed that lysine mutations in nsp12 did not affect its interaction with nsp9 (Fig. 7A). Notably, however, the nsp12^K89/91/127/130^ mutant exhibited enhanced binding to nsp10, while other mutants showed no change in this interaction (Fig. 7B). We also assessed the effect of these nsp12 lysine mutants on the assembly of the replication and transcription complex (RTC). The nsp12 mutants did not affect the nsp10-nsp9 interaction; interestingly, no interaction was detected between nsp11 and nsp10 (Fig. 7C). Given that nsp12 is critical for sgRNA synthesis, we further explored whether these lysine mutations influence PRRSV-2 sgRNA production. We found that rPRRSV-nsp12^R89K^ generated less sgRNA, whereas rPRRSV-nsp12^K91/127/130R^ promoted sgRNA synthesis compared to rPRRSV (Fig. 7D). Collectively, these results indicate that lysine-to-arginine adaptive mutations in nsp12 neither affected its interaction with nsp9 or nsp10 nor the assembly of the RTC, but specifically enhance sgRNA synthesis.

### The nsp12 interactome is primarily associated with ubiquitination-related modifications

**Fig 8:**
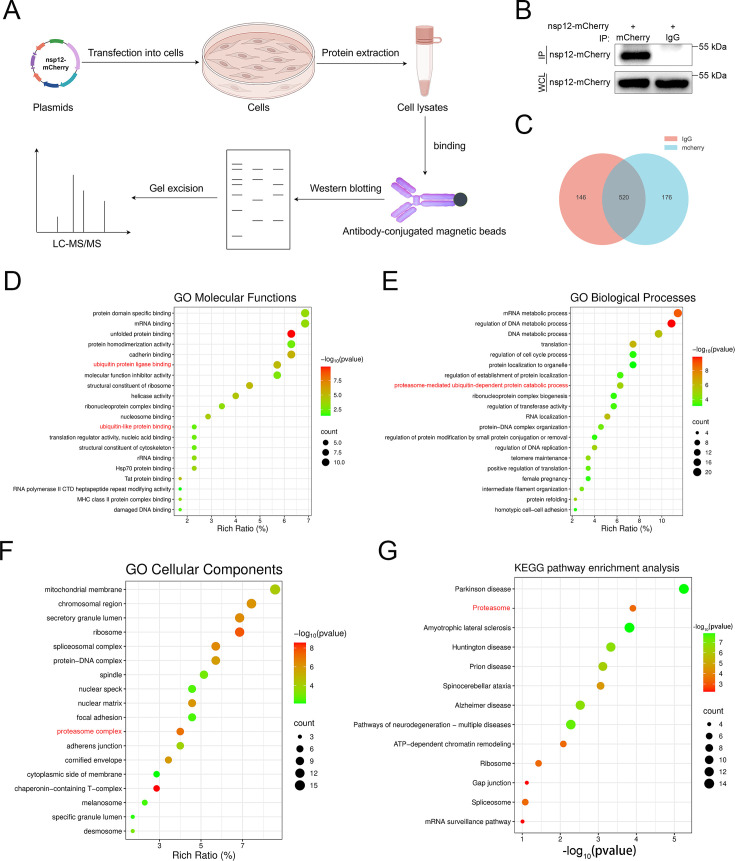
The nsp12 interactome is primarily associated with ubiquitination-related modifications. (**A**) Schematic diagram showing the IP-MS procedure for mapping the nsp12-host protein interactomes. (**B**) HEK293T cells were transfected with plasmids encoding nsp12-mCherry, followed by Co-IP using either anti-mCherry or control anti-IgG conjugated beads. The immunoprecipitated complexes were analyzed by both western blotting and LC-MS/MS. (**C**) Identification of proteins specifically interacting with nsp12 by a Venn diagram in Venny. (**D–F**) GO function analysis. (**G**) KEGG pathway enrichment analysis.

To elucidate the interaction network and regulatory mechanisms between nsp12 and host proteins during PRRSV-2 infection, we performed liquid chromatography and tandem mass spectrometry (LC-MS/MS) using nsp12-mCherry as bait (Fig. 8A). Co-IP experiments were conducted to pull down the nsp12-mCherry complex and its interacting proteins from cell lysates using an anti-mCherry antibody. The results showed that the anti-mCherry antibody successfully pulled down the nsp12-mCherry complex, whereas the control anti-IgG antibody failed to do so (Fig. 8B). The immunoprecipitated proteins were subsequently analyzed by mass spectrometry, which identified 176 proteins specifically interacting with nsp12 (Fig. 8C; Files S1 and S2). To explore the biological functions of these interacting proteins, Gene Ontology (GO) analysis was performed, revealing significant enrichment in genes related to ubiquitination modification (Fig. 8D through F). This finding was further corroborated by KEGG pathway analysis, which demonstrated that nsp12-interacting proteins were predominantly associated with ubiquitination pathways (Fig. 8G). These results provide a global landscape of nsp12-host interaction network and highlight that ubiquitination is a core regulatory layer for nsp12 function. Given that most of these interacting proteins have not yet been validated, this interactome data set serves as a valuable hypothesis-generating resource for future research on PRRSV-2 nsp12-mediated regulatory mechanisms.

## DISCUSSION

Post-translational modifications, particularly ubiquitination, are critical regulators of viral replication, immune evasion, and host antiviral responses, making them promising therapeutic targets (29). In PRRSV infection, ubiquitination plays diverse roles: USP1 deubiquitinates nsp1β to stabilize it and enhance viral replication (30), while NLRP12 promotes ubiquitination and degradation of GP2a (31). Although nsps are pivotal for PRRSV replication and virulence (14, 32, 33), the mechanisms by which host-mediated ubiquitination regulates their stability remain unclear. In this study, we found that PRRSV-2 nsp12, a conserved 153-amino-acid protein essential for synthesizing viral sgRNAs (34, 35), is unstable and degraded via the host proteasome pathway. It underwent both K48- and K63-linked polyubiquitination, indicating potential degradation through multiple ubiquitin-dependent pathways. Previous studies have shown that PSMB1-driven degradation of nsp12 relies primarily on its ubiquitination at lysine 130, whereas LGALS3BP recruits Cullin3 E3 ligase via its BACK domain to mediate nsp12 ubiquitination at lysine 91 (36, 37). Our results extend these findings by revealing that PRRSV-2 nsp12 underwent ubiquitination not only at lysine residues 91 and 130 but also at lysine residues 89 and 127, suggesting that additional host factors contribute to the regulation of nsp12 stability. We further demonstrated that RNF114 promotes nsp12 degradation via its E3 ligase activity, primarily targeting K127 and K130. Mutating RNF114’s catalytic site impaired its ability to suppress PRRSV-2 replication, consistent with its role in inhibiting other viruses (28).

RNA viruses, characterized by high mutation rates and recombination capacity, pose substantial challenges for disease control and prevention. To adapt to environmental pressures and evade host defense mechanisms, viral proteins frequently acquire adaptive mutations at specific amino acid residues, thereby enhancing viral fitness and replication (38–40). Here, we investigated whether PRRSV-2 nsp12 employs such adaptive mutations to escape host degradation. Evolutionary analysis revealed a notable trend toward arginine substitution at lysine residues within PRRSV-2 nsp12. Importantly, this adaptive strategy of lysine-to-arginine substitution is unique to PRRSV-2 and not a conserved mechanism shared by PRRSV-1, highlighting a distinct evolutionary adaptation of PRRSV-2 to its host. To functionally validate this observation, we generated a recombinant PRRSV-2 strain carrying lysine-to-arginine mutations at key positions (rPRRSV-nsp12^K91/127/130R^). This strain exhibited enhanced replication capacity *in vitro*. Conversely, the rPRRSV-nsp12^R89K^ strain showed markedly reduced infectivity *in vitro*. These findings suggest that PRRSV-2 escapes host-mediated ubiquitination-dependent degradation by mutating lysine to arginine, thereby enhancing viral fitness. A similar strategy is used by the influenza virus (41). While our *in vitro* studies in Marc-145 cells and PAMs establish the importance of these adaptive mutations for PRRSV-2 replication, further *in vivo* validation in pigs is warranted to confirm their relevance during natural infection.

Nsp12 is essential for sgRNA synthesis (34), and our findings demonstrate that lysine-to-arginine adaptive mutations in PRRSV-2 nsp12 enhance sgRNA production, which likely underpins the observed increase in PRRSV-2 replication. Nsp9, nsp10, and nsp11 are core components of the PRRSV RTC (42), and nsp12 has been shown to interact with nsp9 and nsp10 (43). We initially hypothesized that nsp12 mutations might modulate sgRNA synthesis by altering these protein-protein interactions. However, our results revealed that none of the nsp12 lysine mutants affected its interaction with nsp9. Interestingly, the nsp12^K89/91/127/130R^ mutant exhibited enhanced binding to nsp10, whereas other mutants showed no significant change, suggesting that this strengthened interaction may partially contribute to the enhanced viral replication. Furthermore, nsp12 mutations did not disrupt RTC assembly, as evidenced by the unaltered nsp10-nsp9 interaction. Collectively, these observations indicate that the enhanced sgRNA synthesis driven by nsp12 lysine mutations is independent of RTC integrity and instead correlates with alterations in nsp12 ubiquitination and protein stability. This notion is further supported by mass spectrometry analysis, which revealed enrichment of ubiquitination-related proteins among PRRSV-2 nsp12-interacting partners, although the functional roles of these interactions warrant further study.

**Fig 9:**
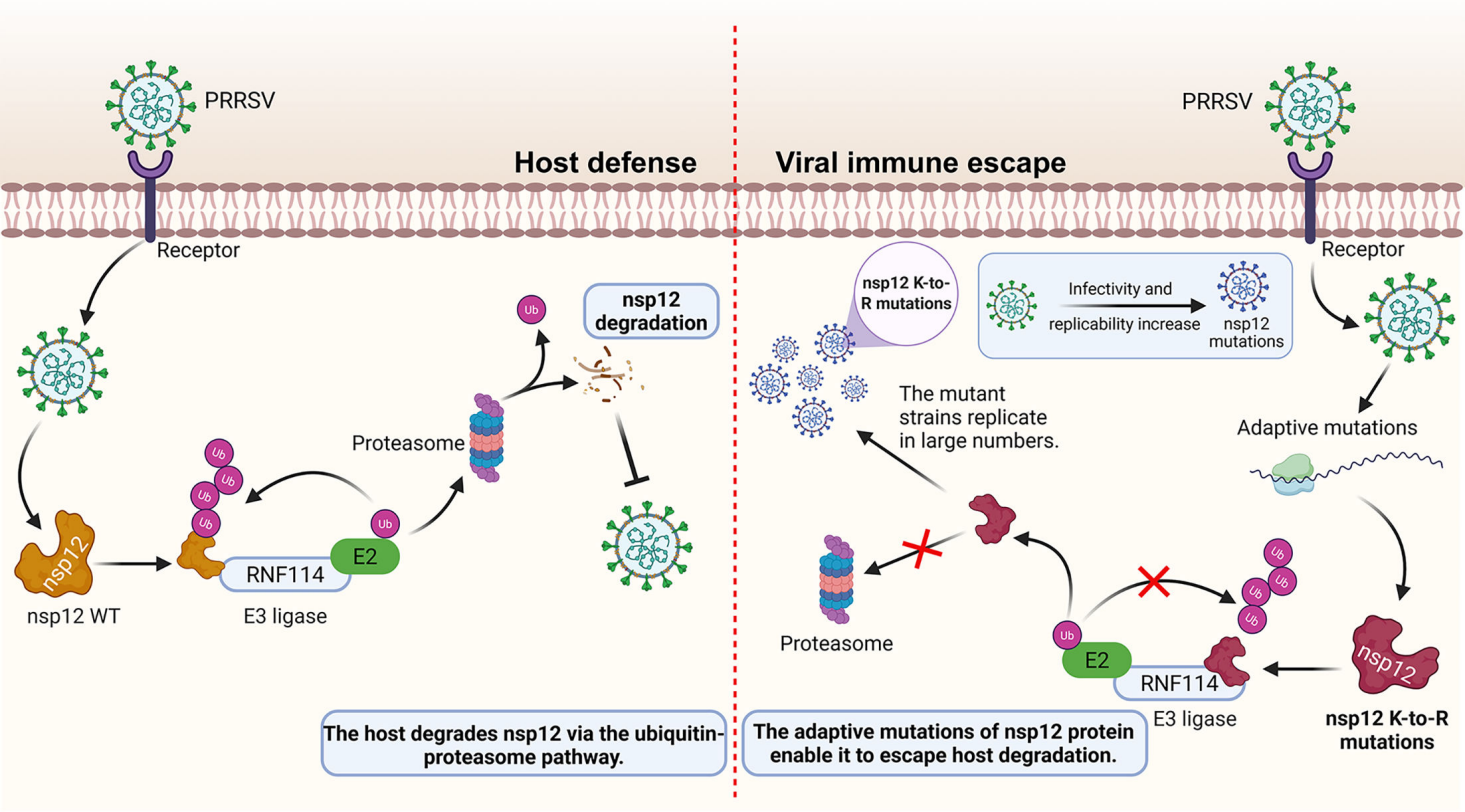
A model demonstrating how adaptive lysine mutations within PRRSV-2 nsp12 evade host proteasomal degradation. Upon PRRSV-2 infection, host E3 ubiquitin ligases specifically interact with viral nsp12 protein and mediate its ubiquitination, leading to subsequent degradation via the proteasome pathway and ultimately restricting viral replication. However, evolutionary pressure has driven the accumulation of arginine substitutions at critical lysine residues within nsp12. These mutations abolish ubiquitination-dependent degradation of nsp12, resulting in enhanced protein stability and increased sgRNA synthesis, thereby promoting viral replication efficiency.

In summary, our findings reveal a host-virus arms race in which the host ubiquitin-proteasome system targets PRRSV-2 nsp12 for degradation, while the virus counteracts this restriction through adaptive lysine-to-arginine mutations that enable immune evasion and enhance viral fitness. We demonstrate that acquisition of non-lysine residues at key sites not only stabilizes nsp12 but also promotes sgRNA synthesis, thereby facilitating viral replication (Fig. 9). These insights advance our understanding of PRRSV-2 pathogenesis and highlight the critical role of adaptive mutations in viral evolution. Furthermore, targeting nsp12 stability or its ubiquitination-mediated degradation may represent a promising strategy for antiviral intervention.

## MATERIALS AND METHODS

### Cells and viruses

African green monkey kidney cells (Marc-145) and HEK293T cells were cultured in Dulbecco’s modified Eagle’s medium (DMEM; Gibco, 8122784) supplemented with 10% fetal bovine serum (FBS; ExCell Bio, FSP500). The 3D4/21 cells (immortalized PAMs) were cultured in RPMI 1640 medium containing 10% FBS. Primary PAMs, the natural target cells for PRRSV infection, were harvested from 4-week-old PRRSV-negative piglets and cultured in RPMI 1640 medium (Gibco) supplemented with 10% FBS. All cells were cultured at 37 °C with 5% CO_2_. PRRSV-2 strains CH-1a (AY032626.1), TA-12 (HQ416720.1), JXA1 (FJ548855.1), SD16 (JX087437.1), and WUH3 (HM853673.2) were preserved in our laboratory. The recombinant strain rHP-PRRSV TA-12 (rPRRSV) was generated using a method similar to that described previously, with minor modifications (44). PRRSV-2 was propagated and titrated on Marc-145 cells.

### Antibodies and reagents

The mouse monoclonal antibody specific to PRRSV N (JN0401) was purchased from MEDIAN Diagnostics (Korea). Anti-PRRSV nsp1α antibody (GTX133695) was purchased from GeneTex. Anti-mCherry-tag (26765-1-AP or 68088-1-Ig), anti-GAPDH (60004-1-Ig), anti-ubiquitin (Ub) (10201-2-AP), and HRP-conjugated anti-mouse (SA00001-1) or -rabbit (SA00001-2) antibodies were purchased from Proteintech Group. Anti-HA-tag (3724) and Alexa Fluor 488-conjugated goat anti-rabbit (4412) or -mouse (4408) antibodies were purchased from Cell Signaling Technology. Chemical reagents MG132 (HY-13259) and cycloheximide (CHX; HY-12320) were purchased from MedChemExpress. 4',6-diamidino-2-phenylindole, dihydrochloride (DAPI; C0065, 1:1,000) was purchased from Solarbio. Protein A/G Magnetic Beads (B23202) were purchased from Selleck Chemicals. Lipofectamine 3000 (Invitrogen, L3000015) was purchased from Thermo Fisher Scientific.

### Plasmid construction and cell transfection

The nsp12 from the PRRSV-2 VR2332 genome was subcloned into the pmCherry-N1 vector to generate the nsp12-mCherry fusion expression plasmid. Nsp12 mutants were generated from nsp12-mCherry by site-directed mutagenesis or chemical synthesis. The nsp12 coding sequence from EAV (DQ846750) was synthesized and cloned into the pmCherry-N1 vector to create an EAV-nsp12-mCherry fusion plasmid. HA-tagged ubiquitin mutants (K6, K11, K27, K29, K33, K48, K63, K48R, and K63R) were generated from HA-Ub through point mutations. Myc-tagged RNF114 was cloned into the pcDNA3.1-Myc vector. All constructed plasmids were verified by Sanger sequencing. For cell transfection, cells grown in culture plates were transfected at 80% confluence using either polyethylenimine (PEI; Servicebio, G1802) or Lipofectamine 3000 at a ratio of 1 µg plasmid to 3 µL transfection reagent.

### MG132 and CHX treatment

For MG132 treatment, HEK293T cells were transfected with plasmids for 12 h before the addition of the inhibitor. After a 12 h incubation, cells were harvested, and lysates were analyzed by immunoblotting. For the protein half-life assay, HEK293T cells were transfected with plasmids for 24 h and then treated with the protein synthesis inhibitor CHX (50 μg/mL). Cells were collected at the indicated time points, and target protein levels were analyzed by western blotting.

### Western blotting and Co-IP assay

For the western blotting assay, cells were lysed on ice for 30 min using IP cell lysis buffer (Beyotime, P0013J) supplemented with 1 mM PMSF (Solarbio, P0100). Proteins were resolved by sodium dodecyl sulfate polyacrylamide gel electrophoresis (SDS-PAGE) and transferred electrophoretically onto polyvinylidene difluoride (PVDF) membranes (Merck Millipore, ISEQ00010). Membranes were blocked with 5% skim milk (Beyotime, P0216) for 2 h at room temperature, followed by overnight incubation at 4°C with primary antibodies and 1 h incubation at room temperature with HRP-conjugated anti-rabbit/mouse secondary antibodies. Protein bands were visualized using a Chemiluminescence Detection Kit (MeilunBio, MA0186-3).

For the Co-IP assay, magnetic beads were conjugated to specified antibodies via 30 min of incubation at room temperature. Cell lysate supernatants were incubated with antibody-conjugated beads for 2 h at room temperature or overnight at 4°C. Bead-bound complexes were washed three times, transferred to fresh tubes, and washed three additional times. Proteins were eluted by boiling in protein loading buffer for western blotting analysis.

### Indirect immunofluorescence assay

After washing with PBS, the cells were fixed with 4% paraformaldehyde (Beyotime, P0099) for 15 min at room temperature, permeabilized with 0.5% Triton X-100 (Beyotime, P0096) for 5 min, and blocked with 1% bovine serum albumin (BSA; Beyotime, ST2249) for 1 h at room temperature. Cells were then incubated overnight at 4°C with primary antibodies. After washing to remove unbound primary antibodies, cells were incubated with appropriate fluorescently labeled secondary antibodies for 1 h at room temperature. Following secondary antibody removal and washing, nuclei were counterstained with DAPI for 5 min. Fluorescence images were acquired using either an inverted fluorescence microscope (Nikon ECLIPSE Ti2) or a confocal laser scanning microscope (Leica, SP8).

### RNA interference (RNAi) assay

Small-interfering RNAs (siRNAs) targeting pig RNF114, along with scramble siRNA controls, were synthesized by IGE biotech (Guangzhou, China). PAMs were transfected with siRNAs for 24 h using Lipofectamine 3000, followed by infection with the PRRSV-2 JXA1 strain for 24 h. The siRNA sequences are listed in Table S3.

### Phylogenetic and sequence analyses of the PRRSV-2 nsp12

For phylogenetic analysis, a neighbor-joining (NJ) method in MEGA software was used to construct phylogenetic trees with 1,000 bootstrap replicates. Tree visualization, manipulation, and annotation were performed using the Interactive Tree of Life platform (iTOL; https://itol.embl.de). For sequence analysis, nucleotide and amino acid homology of nsp12 was analyzed with the Clustal W method in the MegAlign program of the DNASTAR software package (version 7.0; DNASTAR, Madison, WI). Sequence alignment and site-specific variation analysis of nsp12 amino acid sequences were conducted using BioEdit software (version 7.2.6.1). The frequency of lysine mutations in PRRSV-2 nsp12 was counted using WebLogo3 (http://weblogo.berkeley.edu/).

### RNA extraction and reverse transcription quantitative PCR (RT-qPCR)

Total RNA was extracted using VeZol Reagent (Vazyme, R411) and reverse-transcribed into complementary DNA (cDNA) using the HiScript II Q RT SuperMix for qPCR Kit (Vazyme, R223). Quantitative real-time PCR was performed with the ChamQ Blue Universal SYBR qPCR Master Mix (Vazyme, Q312) on a QuantStudio 3 instrument (Applied Biosystems, USA). Gene expression changes were calculated via the 2^-ΔΔCt^ method using *GAPDH* (for Marc-145 or HEK293T cells) or *HPRT1* (for PAMs) as the endogenous control. All primer sequences are listed in Table S4.

### Plaque assay

Marc-145 cells were cultured in six-well plates until reaching 100% confluence. The cells were infected with a 10-fold serial dilution of PRRSV for 1 h, with gentle mixing every 15 min. After infection, the culture medium was removed, and cells were washed twice with PBS. Subsequently, cells were overlaid with DMEM containing 1% low-melting agarose (Sangon Biotech, A600015) and 2% FBS. After agarose solidification at room temperature, plates were inverted and incubated at 37°C with 5% CO₂. At 72 h after infection, the agarose layer was carefully removed, and the plaques were visualized after staining with crystal violet solution.

### LDH release assay

Marc-145 cells were cultured in a 12-well plate and infected with PRRSV-2. Cell culture supernatants were collected at 12, 24, 36, 48, 60, and 72 h. LDH release was measured using the CytoTox 96 Non-Radioactive Cytotoxicity Assay (Promega, G1780) according to the manufacturer’s instructions.

### Construction and rescue of recombinant viruses

The highly pathogenic rTA-12 strain was used as the backbone to construct recombinant viruses carrying the K91/127/130R or R89K mutations in nsp12, following a procedure similar to that previously described (44). Briefly, HEK293T cells were cultured in six-well plates to 80% confluence, then transfected with 2.5 μg of infectious full-length cDNA clones using Lipofectamine 3000. At 48 h post-transfection, culture supernatants were collected as passage zero (P0) virus and used to infect Marc-145 cells. When CPE became evident, both cells and supernatants were harvested as P1 virus and subjected to freeze-thaw cycles for propagation.

### Functional enrichment analysis of interacting proteins

Functional annotation of nsp12-interacting proteins was performed through GO term enrichment using the Metascape website (https://metascape.org/gp/index.html), while the KEGG pathway analysis was conducted using DAVID Bioinformatics Resources (https://davidbioinformatics.nih.gov). The enriched functional terms were then analyzed and visualized using an online bioinformatics platform (https://www.bioinformatics.com.cn/).

### Statistical analysis

All data were analyzed using GraphPad Prism 8 software. The differences between the two groups were assessed using Student’s *t*-test, while one-way or two-way analysis of variance (ANOVA) was used for multiple comparisons. A *P*-value < 0.05 was considered statistically significant (*P < 0.05, **P < 0.01, ***P < 0.001), and “ns” indicates no significant difference.

## Data Availability

All data supporting the conclusions are included in this article and its supplemental material. Other source data provided by our findings are also available from the corresponding author upon reasonable request.

## SUPPLEMENTAL MATERIAL

Supplemental material jvi.00479-26-s0001.docx Fig. S1 to S6; Tables S1 to S4.

Files S1 and S2 jvi.00479-26-s0002.xlsx Details of the proteins pulled down by anti-mCherry and anti-IgG conjugated beads.

ASM does not own the copyrights to Supplemental Material that may be linked to, or accessed through, an article. The authors have granted ASM a non-exclusive, world-wide license to publish the Supplemental Material files. Please contact the corresponding author directly for reuse.
