# Supplementary material for: Adaptive mutations at lysine residues of PRRSV-2 nsp12 enable evasion of host proteasomal degradation to promote subgenomic RNA synthesis
Source: J Virol. 2026 Jun 10;100(7):e00479-26. doi: 10.1128/jvi.00479-26 (PMC13386911; doi:10.1128/jvi.00479-26)
Supplement: Supplemental material — Fig. S1 to S6; Tables S1 to S4. [file jvi.00479-26-s0001.docx]

Supplementary Materials for

**Adaptive mutations at lysine residues of PRRSV-2 nsp12 enable evasion of host proteasomal degradation** **to promote subgenomic RNA synthesis**

Yongjie Chen ^1^, Zishen Chen ^1^, Ling Huang ^1^, Siyong Zeng^1^, Baoying Huang ^1^, Chunhe Guo ^1,^*

*Corresponding author. Email: guochunh@mail.sysu.edu.cn

This file includes Figures S1 to S6 and Tables S1 to S4.

**Supplementary figures and figure legends**

**Figure S1**

**
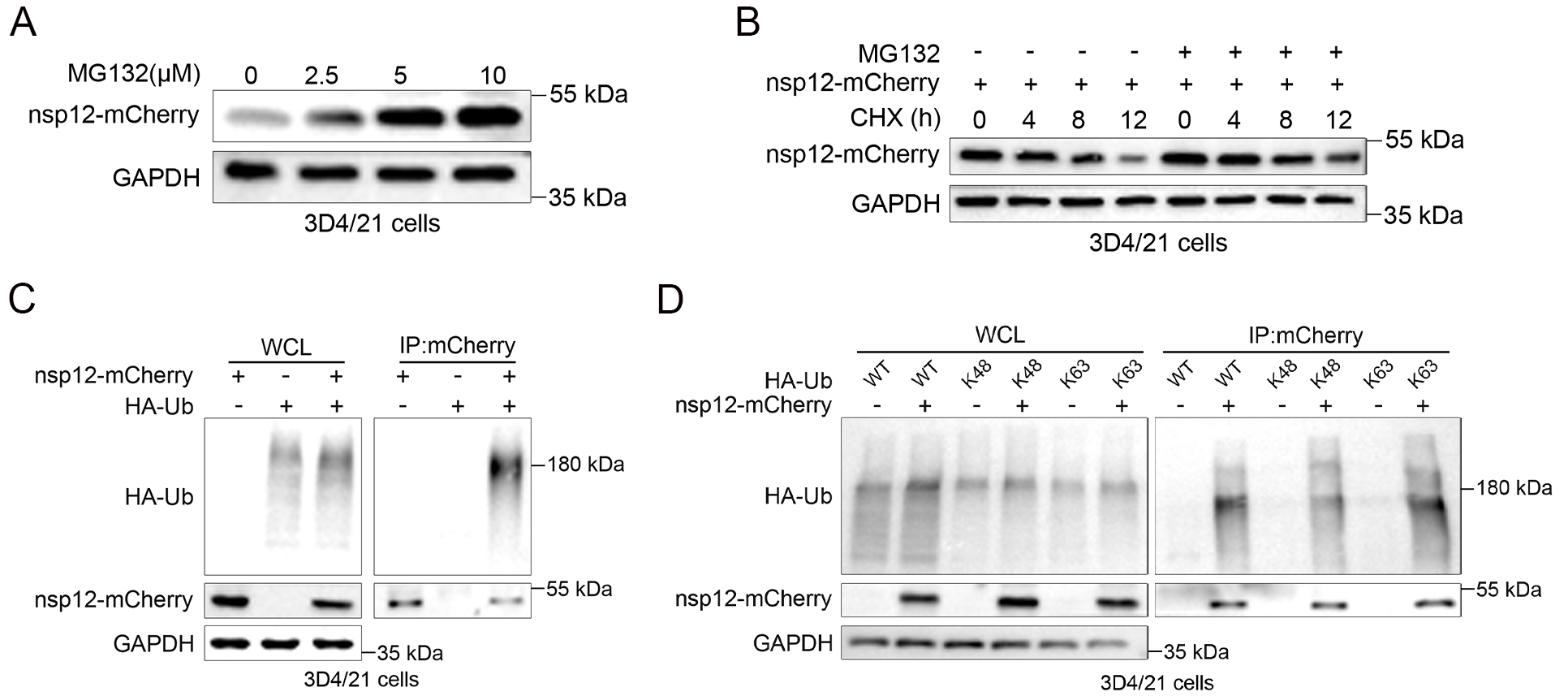
**

**Figure S1. PRRSV-2 nsp12 was degraded via the ubiquitin-proteasome pathway in 3D4/21 cells.** (A) 3D4/21 cells were transfected with nsp12-mCherry and treated with different concentrations of MG132 for 12 h prior to harvesting for western blotting. (B) 3D4/21 cells were transfected with nsp12-mCherry for 12 h, treated with or without MG132 (10 μM) for 12 h, followed by CHX (50 μg/mL) addition. Cells were harvested at indicated time points and lysates were analyzed by western blotting. (C) 3D4/21 cells were transfected with nsp12-mCherry or empty vector, together with HA-Ub or empty vector. Co-IP was performed, followed by western blotting. (D) 3D4/21 cells were transfected with nsp12 and Ub-WT or Ub mutants, followed by Co-IP and western blotting. All experiments were repeated at least three times with independent biological replicates.

**Figure S2**


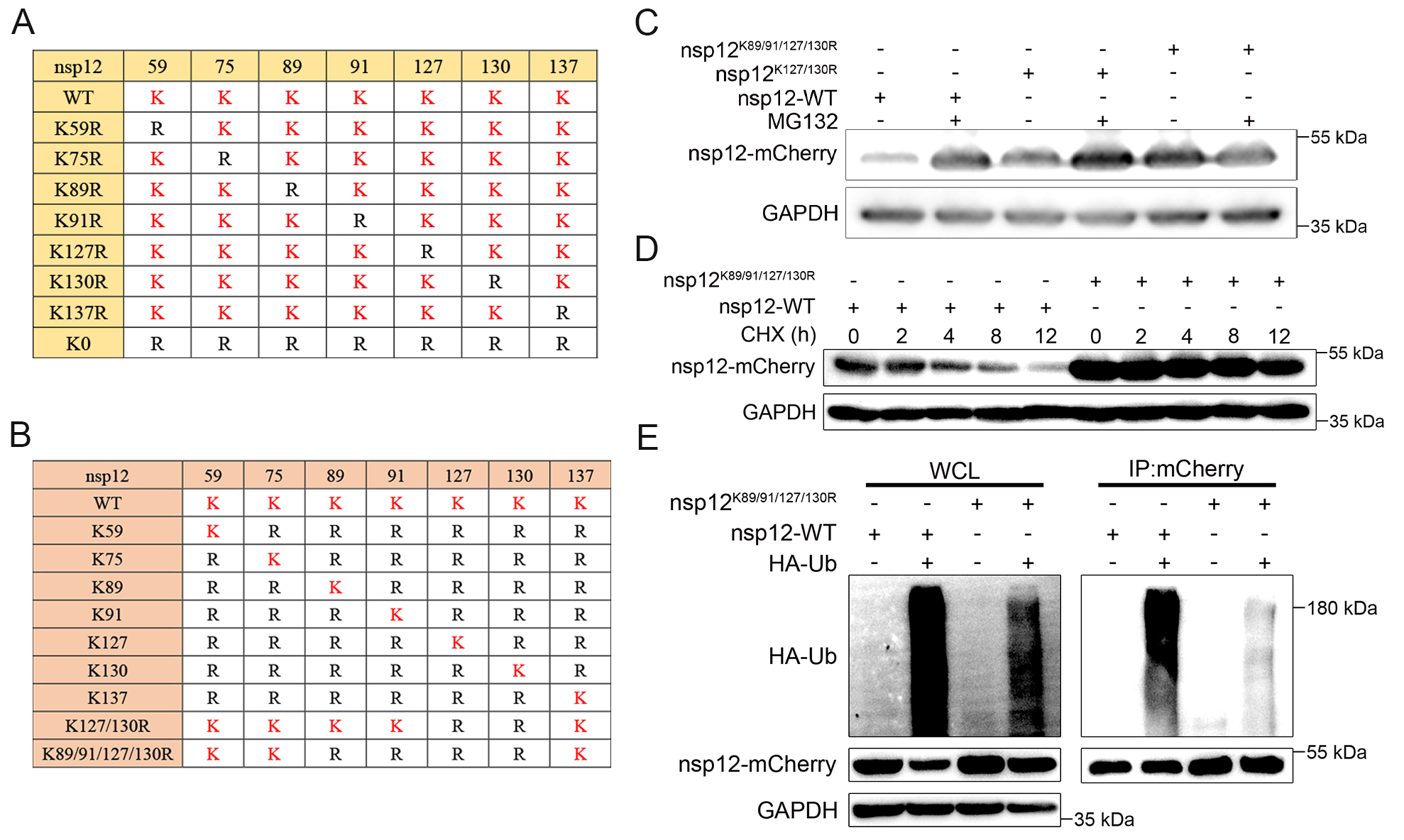


**Figure S2. The lysine residues K89, K91, K127, and K130 serve as the primary ubiquitination sites on nsp12.** (A) Schematic diagram of the construction strategy for nsp12 lysine-to-arginine mutations. (B) Schematic diagram illustrating the use of the K0 mutant as a backbone, restoring arginine to lysine. (C) HEK293T cells were transfected with plasmids expressing nsp12^K127/130R^ or nsp12^K89/91/127/130R^. Cells were treated with or without MG132 (10 μM) for 12 h prior to harvest and analysis by western blotting. (D) HEK293T cells were transfected with nsp12^WT^ or nsp12^K89/91/127/130R^ for 24 h, followed by the addition of CHX (50 μg/mL). Cells were harvested at different time points, and cell lysates were analyzed. (E) HEK293T cells were cotransfected with the indicated nsp12 constructs and HA-Ub (or empty vector), then subjected to Co-IP and western blotting. All experiments were repeated at least three times with independent biological replicates.

**Figure S3**


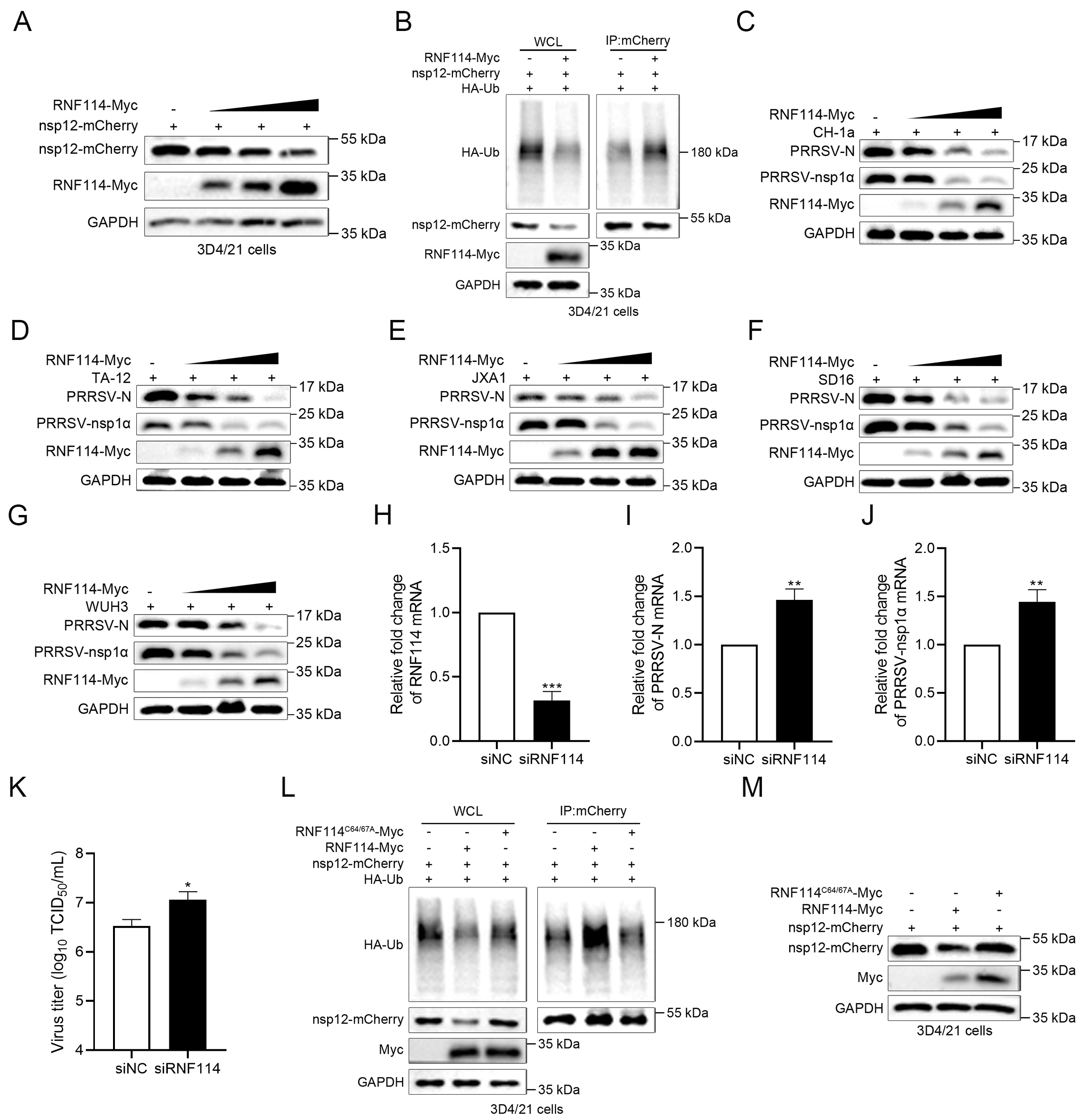


**Figure S3.** **The ubiquitin ligase activity of RNF114 is essential for both nsp12 degradation and viral replication.** (A) 3D4/21 cells were cotransfected with nsp12 and increasing amounts of RNF114, followed by western blotting. (B) 3D4/21 cells were cotransfected with nsp12, HA-Ub, and either RNF114 or empty vector, followed by Co-IP with anti-mCherry beads. (C-G) Marc-145 cells were transfected with Myc-tagged empty vectors or Myc-tagged RNF114 plasmids for 24 h and then infected with multiple PRRSV-2 strains (CH-1a, TA12, JXA1, SD16, and WUH3) at an MOI of 0.5 for 24 h. Viral N and nsp1α protein levels were measured by western blotting. (H-K) PAMs were transfected with siNC or siRNF114 for 24 h, then infected with PRRSV-2 JXA1 at an MOI of 0.5 for an additional 24 h. Viral N and nsp1α mRNA levels were measured by RT-qPCR (H-J), and viral titers in supernatants were quantified by TCID_50_ assay in Marc-145 cells (K). (L) 3D4/21 cells were cotransfected with nsp12, HA-Ub, and either RNF114 or RNF114^C64/67A^. Co-IP was performed using anti-mCherry beads. (M) 3D4/21 cells were cotransfected with nsp12 and either RNF114 or RNF114^C64/67A^, followed by western blotting. All experiments were repeated at least three times with independent biological replicates. Data are shown as mean ± SD (n = 3). *: *P* < 0.05; **: *P* < 0.01; ***: *P* < 0.001; ns: not significant (Student’s t-test).

**Figure S4**

**
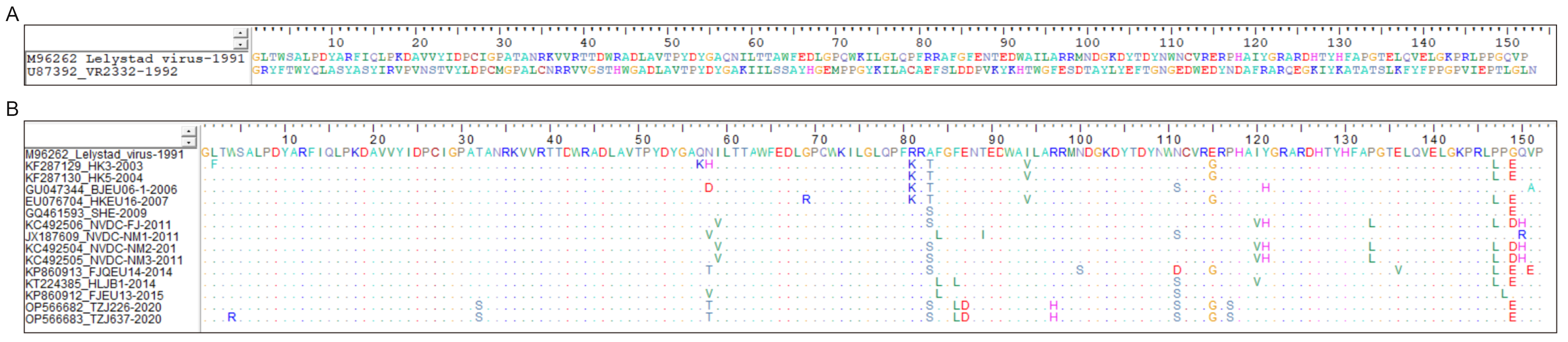
**

**Figure S4. Amino acid homology analysis of nsp12.** (A) Amino acid sequence homology analysis of nsp12 between PRRSV-2 strain VR2332 and PRRSV-1 strain Lelystad. (B) Amino acid sequence alignment of nsp12 from different PRRSV-1 strains.

**Figure S5**

**
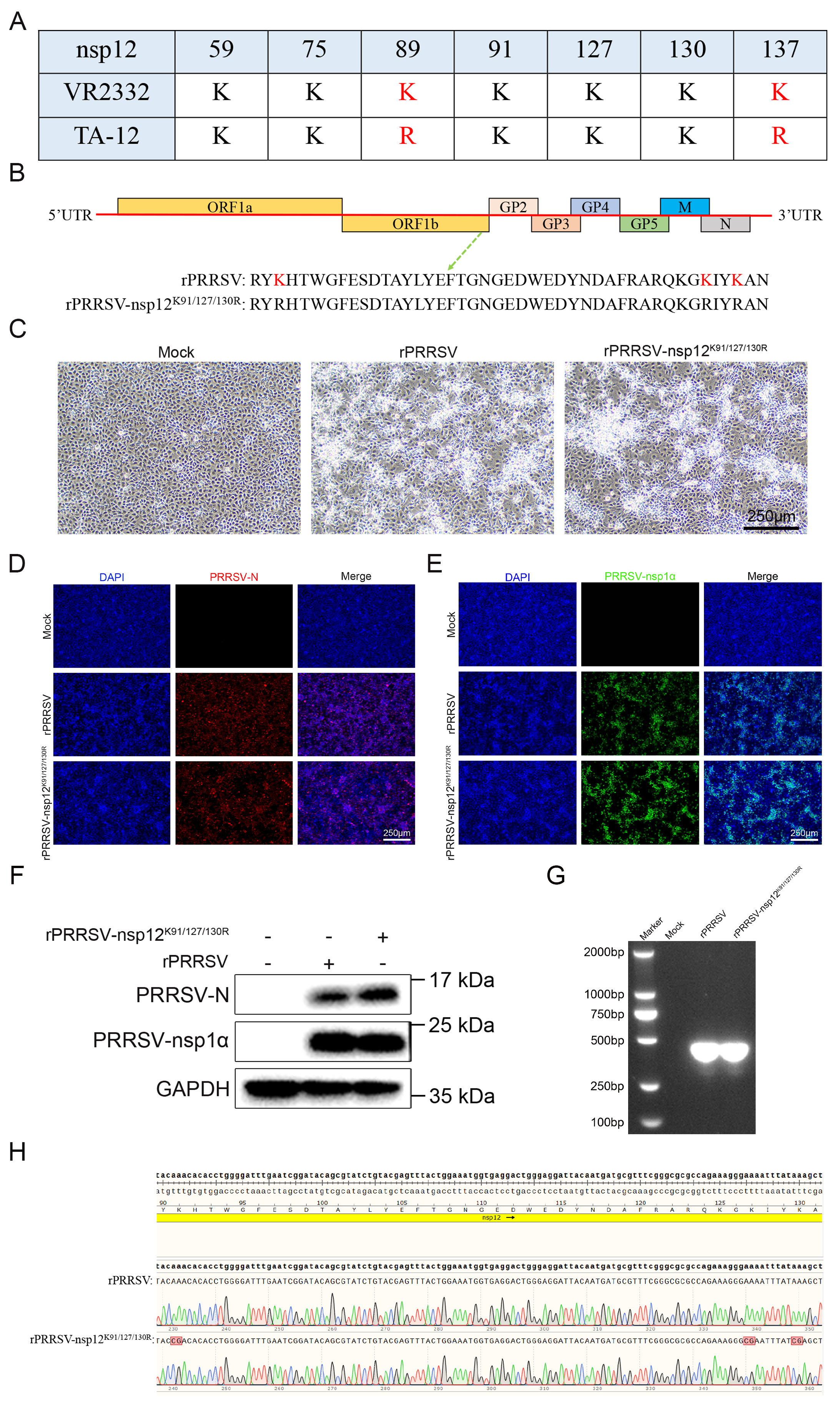
**

**Figure S5.** **Construction and rescue of recombinant strains with lysine-to-arginine mutation at positions 91, 127, and 130 within nsp12.** (A) Comparison of lysine residues at positions 59, 75, 89, 91, 127, 130, and 137 between the VR2332 and TA-12 strains. (B) Schematic diagram illustrating the construction of the rPRRSV-nsp12^K91/127/130R^ strain. (C-H) At 48 h post-transfection of the infectious cDNA clone into HEK293T cells, the culture supernatants were collected and used to infect Marc-145 cells for three days. CPE was observed under a light microscope (C). The presence of PRRSV was confirmed by IFA (D and E) and western blotting targeting N and nsp1α proteins (F). Total RNA was extracted and reverse-transcribed into cDNA. Rescued recombinant strains were identified by RT-PCR (G) and further validated by Sanger sequencing (H).

**Figure S6**


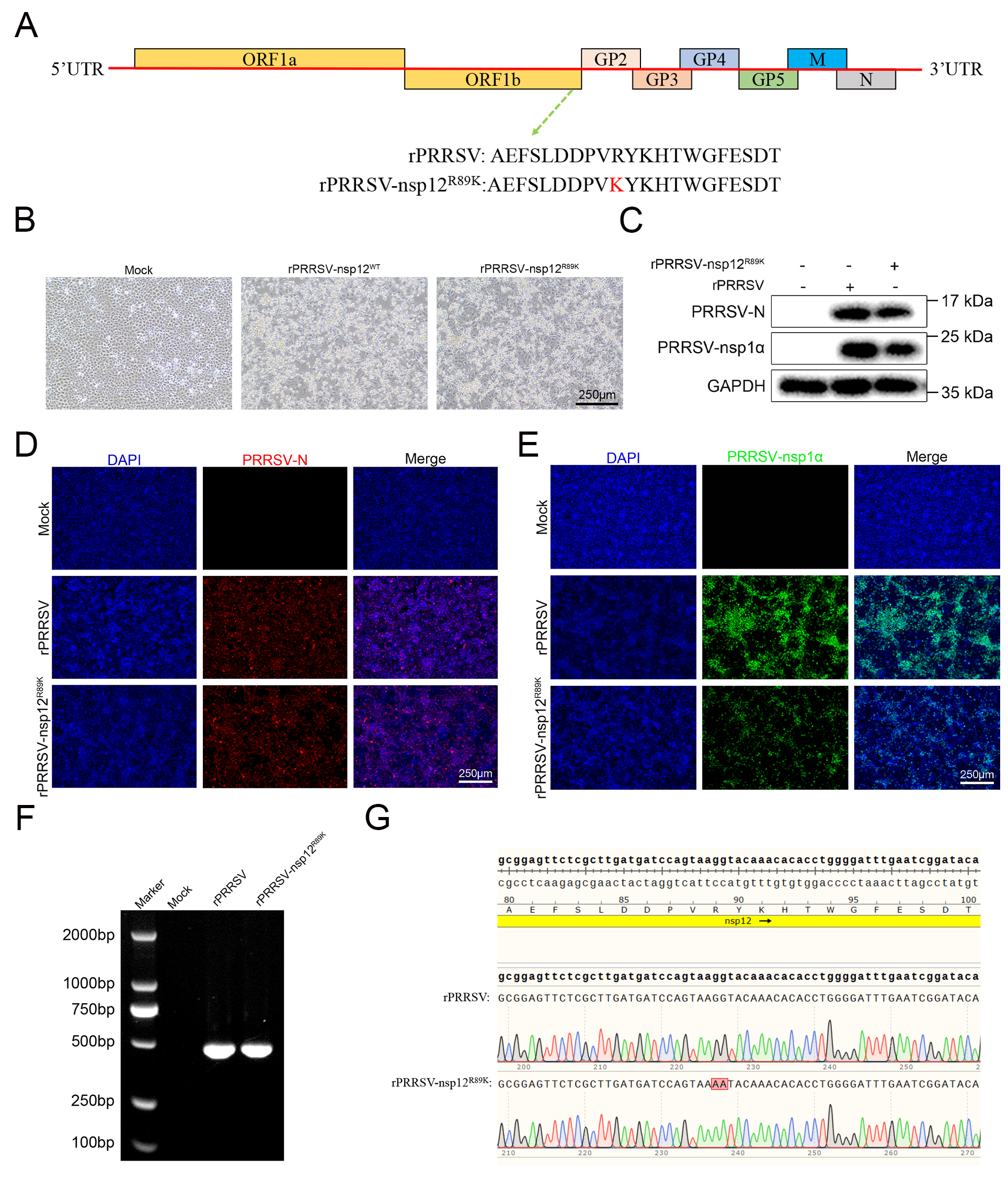


**Figure S6.** **Construction and rescue of TA-12 recombinant strains with arginine-to-lysine mutation at position 89 within nsp12.** (A) Schematic diagram illustrating the construction of the rPRRSV-nsp12^R89K^ strain. (B-G) At 48 h post-transfection of the infectious cDNA clone into HEK293T cells, the culture supernatants were collected and used to infect Marc-145 cells for three days. CPE was observed under a light microscope (B). The presence of PRRSV was confirmed by western blotting (C) and IFA (D and E) targeting the N and nsp1α proteins. Total RNA was extracted and reverse-transcribed into cDNA. Rescued recombinant strains were identified by RT-PCR (F) and further validated by Sanger sequencing (G).

**Supplementary tables**

**Table S1.** PRRSV-2 strain information for the nsp12 reference sequence.

| Year | Region | Strain | Accession no. |
| --- | --- | --- | --- |
| 1992 | USA | ATCC VR2332 | U87392 |
| 1996 | China | CH-1a | AY032626 |
| 1996 | China | BJ-4 | AF331831 |
| 2003 | China | JA142 | AY424271 |
| 2003 | China | HN1 | AY457635 |
| 2006 | China | TJ | EU860248 |
| 2006 | China | JXA1 | EF112445 |
| 2006 | China | HuN4 | EF635006 |
| 2007 | China | GD | EU825724 |
| 2007 | China | WUH1 | EU187484 |
| 2008 | China | XH-GD | EU624117 |
| 2008 | China | CH-1R | EU807840 |
| 2008 | USA | NADC30 | JN654459 |
| 2008 | USA | NADC31 | JN660150 |
| 2009 | China | WUH2 | EU678352 |
| 2010 | China | JX | JX317649 |
| 2011 | China | GM2 | JN662424 |
| 2011 | China | QYYZ | JQ308798 |
| 2012 | China | FJFS | KP998476 |
| 2013 | China | JL580 | KR706343 |
| 2013 | China | FJW05 | KP860911 |
| 2013 | China | HENAN-XINX | KF611905 |
| 2013 | China | HLJB1 | KT351740 |
| 2014 | China | FJ1402 | KX169191 |
| 2014 | USA | IA/2014/NADC34 | MF326985 |
| 2015 | China | HNyc15 | KT945018 |
| 2015 | China | GDsg | KX621003 |
| 2016 | China | HNhx | KX766379 |
| 2016 | China | JXwn06 | EF641008 |
| 2017 | China | LNWK96 | MG860516 |
| 2017 | China | SCcd17 | MG914067 |
| 2017 | China | FJM4 | KY412888 |
| 2018 | China | SCya18 | MK144543 |
| 2018 | China | HBap4-2018 | MZ579701 |
| 2019 | China | NL1207 | MZ399800 |
| 2020 | China | SXSZ-2020 | MW880772 |
| 2021 | China | GD-H1 | ON691480 |
| 2021 | China | SDWH86 | OQ506516 |
| 2021 | China | XJSW-2021 | OR247780 |
| 2022 | China | CHjs2022 | PP053518 |
| 2023 | China | SDVD-NMG2023 | PQ644630 |
| 2024 | China | GDZH2024 | PQ462371 |

**Table S2.** The frequency of lysine mutations at positions 59, 75, 89, 91, 127, 130 and 137 within nsp12 of different PRRSV-2 strains.

| Nsp12 | | 59 | 75 | 89 | 91 | 127 | 130 | 137 |
| --- | --- | --- | --- | --- | --- | --- | --- | --- |
| **Sublieage 8.7** | XH-GD-2008 | K | K | R | K | K | K | R |
|  | JXwn06-2016 | K | K | R | K | K | K | R |
|  | JXA1-2006 | K | K | R | K | K | K | R |
|  | TJ-2006 | K | K | R | K | K | K | R |
|  | XJSW-2021 | K | K | R | K | K | K | R |
|  | HuN4-2006 | K | K | R | K | K | K | R |
|  | WUH1-2007 | K | K | R | K | K | K | R |
|  | WUH2-2009 | K | K | R | K | K | R | R |
|  | GD-2007 | K | K | R | K | K | K | R |
|  | JX-2010 | K | K | R | K | K | K | R |
|  | FJ1402-2014 | K | K | R | K | K | K | R |
|  | FJW05-2013 | K | K | R | K | K | K | R |
|  | HLJB1-2013 | K | K | R | K | K | K | K |
|  | HBap4-2018 | K | K | R | K | K | K | R |
|  | SDWH86-2021 | K | K | R | K | K | K | R |
| **Sublieage 8.1** | CH-1a-1996 | K | K | R | K | K | K | K |
|  | CH-1R-2008 | K | K | R | K | K | K | K |
| **Sublieage 1.8** | JA142-2003 | K | K | R | K | K | K | R |
|  | GD-H1-2021 | K | K | R | K | K | R | R |
|  | SDVD-NMG-2023 | K | K | R | K | K | R | R |
|  | CHjs-2022 | Q | K | R | K | K | R | R |
|  | GDZH-2024 | K | K | R | K | K | R | R |
|  | SXSZ-2020 | K | K | R | K | K | R | R |
|  | HNhx-2016 | K | K | R | K | K | R | R |
|  | NL1207-2019 | K | K | R | K | K | R | R |
|  | NADC30-2008 | K | K | R | K | K | R | R |
|  | HENAN-XINX-2013 | K | K | R | K | K | R | R |
|  | SCcd17-2017 | K | K | R | K | K | R | R |
|  | HNyc15-2015 | R | K | R | K | K | R | R |
|  | JL580-2013 | K | K | R | K | K | R | R |
|  | FJM4-2017 | K | K | R | K | K | R | R |
|  | SCya18-2018 | R | K | R | K | K | K | R |
| **Sublieage 1.5** | NADC31-2008 | R | K | R | K | R | K | K |
|  | IA/2014/NADC34-2014 | R | K | R | K | R | K | K |
|  | LNWK96-2017 | R | K | R | K | R | K | K |
| **Sublieage 5.1** | VR2332-1992 | K | K | K | K | K | K | K |
|  | BJ-4-1996 | K | K | K | K | K | K | K |
|  | HN1-2003 | K | K | K | K | K | K | K |
| **Sublieage 3.5** | FJFS-2012 | K | K | R | K | K | K | K |
|  | GDsg-2015 | K | K | R | K | K | K | K |
|  | GM2-2011 | K | K | R | K | K | K | K |
|  | QYYZ-2011 | K | K | R | K | K | K | K |

**Table S3.** Sequences of siRNAs used in this study.

| siRNA | Direction | Sequence (5’-3’) |
| --- | --- | --- |
| siRNF114 | sense | CCAUAGCACGGACACCAAATT |
|  | antisense | UUUGGUGUCCGUGCUAUGGTT |

**Table S4.** Primer sequences used in this study.

| Primer name^a^ | Sequence (5’-3’) |  |
| --- | --- | --- |
| PRRSV-N-F | AAAACCAGTCCAGAGGCAAG |  |
| PRRSV-N-R | CGGATCAGACGCACAGTATG |  |
| PRRSV-nsp1α-F | ACACGATGCCTCAGTGCA |  |
| PRRSV-nsp1α-R | CTCTTCGGGCCTGTAGAAT |  |
| mGAPDH-F | TGACAACAGCCTCAAGATCG |  |
| mGAPDH-R | GTCTTCTGGGTGGCAGTGAT |  |
| pRNF114-F | CCATAGCACGGACACCAAATC | |
| pRNF114-R | CTTCGTCAACGTCGTAATCCAC | |
| pHPRT1-F | TGGAAAGAATGTCTTGATTGTTGAAG | |
| pHPRT1-R | ATCTTTGGATTATGCTGCTTGACC | |
| gRNA-F | CTCCACCCCTTTAACCATGTC | |
| gRNA-R | AATGCACGTGGCAACGTCCAC | |
| sgRNA2-F | CTCTCCACCCCTTKAACCAACTTT | |
| sgRNA2-R | CGGAGCAAACCAGTCTGATGC | |
| sgRNA3-F | CTCCACCCCTKTAACCATAGTG | |
| sgRNA3-R | CCCCTAACCAGCGGAAACCA | |
| sgRNA4-F | CTCCACCCCTTTMACCTGGAA | |
| sgRNA4-R | TGAGGACTTTTGCGAATCGTCG | |
| sgRNA5-F | CTCCACCTTTARCCTGTCT | |
| sgRNA5-R | CCAATCTGTGCCATTCAGCTC | |
| sgRNA6-F | CTCCACCCCTTTAACCAGAGTTT | |
| sgRNA6-R | CTCCACCCCTTTAACCAGAGTTT | |
| sgRNA7-F | CTCCACCCCTWTAACCACGCAT | |
| sgRNA7-R | ACCCAGCATTTGGCACAGCT | |
| hGAPDH-F | AAGGTCGGAGTCAACGG | |
| hGAPDH-R | GGAAGATGGTGATGGGATT | |

^a^ Primer name prefixes indicate species origin: "h" for human, "m" for monkey, "p" for pig.
